# Collective Electrostatics vs through-Space Interactions: Electronic Properties of Molecules with Multiple Polar Substituents

**DOI:** 10.1021/acsphyschemau.5c00104

**Published:** 2025-11-24

**Authors:** Egbert Zojer

**Affiliations:** Institute of Solid State Physics, NAWI Graz, Petersgasse 16, A-8010 Graz, Austria

**Keywords:** collective electrostatics, through-space interactions, substitution, ionization energy, electron affinity, inductive effect, mesomeric effect

## Abstract

Collective electrostatic has been identified as the single most important factor determining the electronic structure and (electronic) functionality of heterogeneous interfaces. It changes spectroscopically determined quantities like electron binding energies and core-level shifts. Additionally, it results in massive changes of surface potentials and injection barriers in conventional and molecular electronic devices and shifts the electrostatic potential within the channels of porous materials. Collective electrostatics is triggered by the superposition of the electric fields of dipoles, which are arranged in a (semi)­periodic fashion. This raises the questions, which role it plays in individual molecules comprising multiple polar substituents and how collective electrostatics is related to the widely discussed through-space interactions between molecular backbones and polar substituents. Thus, the current manuscript will specifically address the question, whether through-space interactions can be regarded as yet another manifestation of collective electrostatics. To that aim, first a model-system is designed in which through-bond interactions with substituents are essentially eliminated. Subsequently, the localization of the encountered frontier orbitals and charging induced polarization effects are studied. Additionally, the evolution of ionization energies, electron affinities and the local distribution of the potential shifts with the number of polar substituents are analyzed. The data as a whole suggest that through space interactions can massively change the electronic properties of molecules due to the combined electric field of the polar substituents; still, distinct deviations from the typical characteristics of systems dominated by collective electrostatics are observed. This shows that in molecules one is rather in the realm of cumulative local-field electrostatic effects.

## Graphical abstract

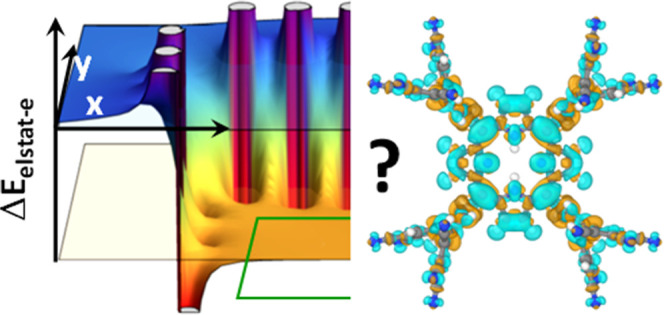

## 1 Introduction

Electron-rich and electron poor substituents are commonly used to change ionization energies and electron affinities of molecules. This is typically associated with the substituents' positive or negative inductive and mesomeric effects. The inductive effect describes the displacement of electrons along σ-bonds due to differences in electronegativity, such that bonds become polarized.^1–3^ In contrast, the mesomeric effect polarizes a molecule by a substitution-induced rearrangement of electrons in the π-system.^4^ Substituents can, however, impact the electronic structure of a molecule not only through bonds but also through space, i.e., via the electric fields produced by polar bonds and polarized atoms. This is sometimes reflected in the use of the term “so-called inductive effect”, which either refers to the field effect alone or to the combined through-bond and through space mechanisms.^5^ Field-related substitution effects have been observed in various contexts dealing, e.g., with reaction kinetics and catalysis.^6–8^ They have also been the subject of multiple theoretical studies.^8–11^ Notably, the relative role played by through-space effects is still under debate, even though their crucial importance becomes more and more apparent.^12,13^

In a seemingly different context, field-induced changes of ionization energies and electron affinities (and the concomitant changes in electronic level alignment) have in the past >20 years been investigated in the field of surface–and interface science. There, one is typically concerned with periodic dipole assemblies generated either by the periodic arrangements of polar molecules or by interfacial charge-transfer processes. Notably, such periodic assemblies of dipoles typically also exist at surfaces of organic thin films.^14^ The resulting effects are then referred to as being caused by collective (or cooperative) electrostatics.^15–21^ This distinguishes the situation from that of fields around individual dipoles, which rapidly decay with distance and, thus, impact the energy landscape only in their immediate vicinity. Notably, also such “near-field” electrostatics changes ionization energies of the molecules the substituents are attached to.^22^ The situation is, however, fundamentally different for extended, 2D dipole layers, as they occur, for example, at interfaces. They induce “global” shifts between the electronic states in the materials below and above the layers^15–21^ with edge effects occurring at their boundaries.^18,23^ Above and below the dipole layers, the “global” shifts decay over length-scales on the same order as the extent of the dipole layers. This can be straightforwardly inferred from a superposition of the electric fields of the individual dipoles arranged in the 2D assemblies.^18,21^ More recently, it has been shown by state of the art simulations that such global shifts are observed also in the cylindrical pores of certain porous framework materials.^24,25^ As a consequence, the electrostatic potential within the pores can be massively changed relative to the potential outside, with the sign of the change depending on the polarity of the substituents. This impacts the alignment between the electronic states in the MOF backbones and the states in guest molecules.^24,25^ Eventually, the above effects lead to the suggestion of the concept of an electrostatic design of materials.^26^ A finding in Ref. 26 of particular relevance for the present paper is that the signature of collective electrostatics (i.e., a shift of the electrostatic energy by an essentially constant value in a specific region of space) can also be triggered by an arrangement of multiple dipoles on the surface of a cube. Overall, the above-described observations suggest that the exact geometrical shape of the dipole assembly appears not to be overly relevant.

This raises the question, what situation would be encountered in a molecule bearing multiple polar substituents arranged in a “dipole-shell”. Such a shell is significantly smaller than any of the systems for which collective electrostatics has been observed so far, but could it still be used to generate a reasonably extended region in space in which a constant, but significantly altered electrostatic energy prevails? To study that, it is crucial to design a molecular system in which the central (π-conjugated) entity is essentially not influenced by the polar substituents in the periphery by through-bond interactions.

## 2 Studied Systems

Such a situation is, for example, realized in the family of molecules shown in Figure 1.

**Figure 1:**
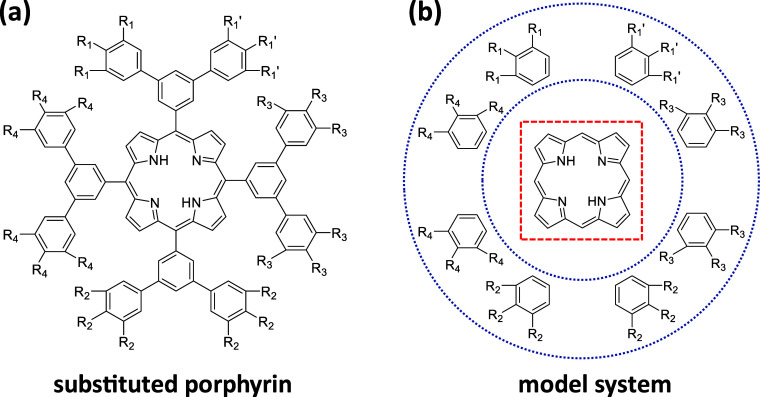
Structure of the studied molecules consisting of a porphyrin core, a set of four “bridging” benzene rings directly attached to the porphyrin, and a second shell of eight “peripheral” benzene rings with two of them bonded to each bridging benzene. The peripheral rings can each be substituted by three polar groups, which in the present study are –F atoms, or –CN and –N­(CH_3_)_2_ groups. For the –F and –CN substituents, the degree of substitution has been varied systematically. For the –N­(CH_3_)_2_ groups only the fully substituted molecule has been calculated to test, whether the sign of the energetic shifts are reversed, when switching from an electron-accepting to a donating substituent. The nomenclature used for the studied molecules shall be exemplarily presented for –F substituents: “1 × (3 F)”: F atoms only at the three substitution positions termed R_1_ in panel (a); “2 × (3 F)” in total six F atoms at positions R_1_ and R_1_′; 4 × (3 F) also R_2_ positions occupied by F;···; “8 × (3 F)” all substituent positions in the eight peripheral rings occupied by F atoms. Panel (b) illustrates a system in which the bridging benzene rings have been removed to eliminate any through-bond coupling between the central porphyrin and the (partially) substituted benzenes in the periphery. When referring to such model systems, the name of the parent molecule will be primed. 3D representations of the molecular structures will be provided in various figures throughout the manuscript.

In the displayed structures, the polar substituents are denoted by –R_1_ to –R_4_, where the different labels are required as in the following simulations also partly substituted molecules will be considered. Regarding substituents, the focus will on polar, primarily π-accepting –CN groups and on σ-accepting –F substituents. To show that equivalent effects (albeit with a switched sign) are to be expected also for electron donating substituents, reference calculations with –N­(CH_3_)_2_ substituents were also performed. The nomenclature used for the different molecules is explained in the caption of Figure 1. The molecules are designed in a dendrimer-like fashion such that due to steric effects the conjugation between the central porphyrin and the substituents is broken: the dihedral angle between the porphyrin plane and the plane of the directly attached, linking benzenes amounts to ∼75° and it is ∼39° between the bridging benzene rings and the peripheral ones (with the quoted values found in the fully –CN substituted molecules and with similar values in the other systems). As a consequence, the planes of the porphyrin and the substituted, peripheral rings are close to perpendicular. Moreover, the peripheral rings and the porphyrins are connected by the bridging benzenes in meta-fashion to further reduce conjugation.

In a gedankenexperiment, to study the elimination of any chemical bonds between the substituents and the conjugated cores, also model systems were considered for which the linking benzenes had been removed (see Figure 1b) keeping the molecular geometry frozen at the one calculated for the regular molecule (for details see next section). In passing it is noted that as a further step, to calculate the shift in electrostatic energies generated by substituted peripheral benzenes, also the porphyrin core was removed, which will become relevant in Section 3.3.

Considering that the molecules studied here are reminiscent of dendrimers, it is worthwhile mentioning that electron-donating and accepting substituents are, indeed, often included in such structures, where they adopt different roles: For example, dendrimers containing (cofacially aligned) donating and accepting entities in their periphery have been synthesized as dyes for thermally activated delayed fluorescence;^27,28^ substituents form polar scaffolds in dendrimers or are positioned at the dendrimer periphery to trap, bind, and encapsulate dye molecules;^29^ dendrimers with an amine surface decoration have been combined with counterions to reduce contact resistances in Si solar cells;^30^ and donor–acceptor systems have been used to tune the optical properties of dendrimers.^31,32^ Of particular interest for the present study is the work by Ren. et al.,^31^ who presented dendrimers based on perylene bisimide cores with polyphenylene dendrons bearing –F and –CN substituents. For these electron affinities up to 3.8 to 3.9 eV were observed in cyclic voltammetry experiments.^31^ Moreover, Kong et al. succeeded in decreasing the electron affinity and especially the ionization energy of dendrimers iteratively including dimethylamines into various generations of dendrons.^33^ Still, none of the mentioned studies and also no study the author is aware of, provides a comprehensive mechanistic explanation for how the electronic states in dendrimers are manipulated by polar substituents of the type illustrated in Figure 1. Thus, this is the focus of the present manuscript.

### 2.1 Theoretical Methodology

All simulations were performed using Gaussian 16, Revision A.03 employing default settings.^34^ To describe the electronic structure of the molecules, the B3LYP functional^35^ as implemented in Gaussian was used. For the unsubstituted molecule, for 8 × (3 F), and for 8 × (3-CN), the impact of including the D3 version of Grimme’s long-range dispersion correction was tested.^36^ This, had virtually no impact on calculated ionization energies and electron affinities and their changes through substitution: the values of the IEs changed by at most 0.03 eV and those of the EAs by at most 0.006 eV. The resulting variations in ΔIE and ΔEA were below 0.01 eV. Therefore, all results described in the following have been obtained without an explicit long-range dispersion correction. For expanding the Kohn–Sham orbitals, the following basis-sets were employed: A 6-31G­(d,p)^37,38^ basis set for the geometry optimization and a 6-311+G­(d,p)^38–40^ basis for single point calculations on neutral and charged species to calculate energies, energy differences, potential distributions, charge rearrangements, and orbitals. Considering that the largest considered molecule contained 182 atoms these basis sets are reasonably large and for some of the ions obtaining convergence was challenging. The problems could be overcome by, e.g., intermediate-step simulations with slightly smaller basis sets, whose densities were then used as starting points for calculations with the final, large basis sets. For all simulations reported below, the equilibrium geometries of the neutral molecules were employed. Thus, the reported IEs and EAs correspond to vertical quantities.

To assess the impact of lattice relaxations, for the unsubstituted molecule, for 8 × (3 F), and for 8 × (3-CN) also the geometries of the ions were relaxed and adiabatic IEs and EAs were calculated employing exactly the same approach as described above for the vertical quantities. Notably, adiabatic here refers only to including the inner-sphere (also referred to as intramolecular) reorganization energy without considering any polarization and relaxation of the possible surroundings of the molecules. Overall, the calculated impact of the inner relaxation was found to be extremely small amounting to 36 (65) meV, 34 (71) meV, and 32 (9) meV for the IEs (EAs) of the unsubstituted molecule, of 8 × (3 F), and of 8 × (3-CN), respectively. A more detailed discussion and the results of simulations on the porphyrin core can be found in the Supporting Information. The particularly low inner-sphere reorganization energy of porphyrin derivatives is consistent with findings both in experiments on isolated molecules^41^ and in simulations of porphyrin derivatives.^42^ The only minor reorganization is often attributed to the particularly stable π-electron system of the porphyrin ring. Moreover, in the present case a further reduction of the inner-ring reorganization energy is potentially caused by the frontier states extending also onto neighboring rings (see below). Consequently, there are essentially no differences in the substitution-induced changes in IE and EA between vertical and adiabatic cases. Only for the anion-formation in 8 × (3-CN) vs the neutral molecule there is a minor difference, but this can be associated with the particularly delocalized charges in this case, which will be discussed below.

As a final technical detail it is mentioned that the geometries for the model systems lacking the bridging benzenes are based on those of the parent systems optimizing only the positions of the H atoms that had to be added to saturate the bonds.

## 3 Results and Discussion

### 3.1 Orbital Localization

As a first indication that the frontier orbitals are quantum-mechanically decoupled from the substituents, they ought to be localized on the central porphyrins. Thus, analyzing the orbitals provides useful first insights, even though ionization energies and electron affinities will later not be calculated from orbital energies, but from energy differences for the removal/addition of an electron. Additionally, the actual charge rearrangements upon ionization including polarization effects will be discussed in Section 3.4. For the orbitals, the desired localization largely occurs for the highest occupied orbitals (HOMOs) of all considered fully and partially –F and –CN substituted systems as well as in the fully -NH_3_-substituted case. The orbitals only slightly extend onto the bridging benzene rings directly attached to the porphyrin. This is exemplarily shown in Figure 2a for the fully –F substituted molecule and in Figure 2c for the –CN substituted system bearing four oppositely arranged (3-fold) –CN substituted rings (i.e., for 4 × (3-CN)). The situation for all molecules is shown in Figures S1–S3 in the Supporting Information.

**Figure 2:**
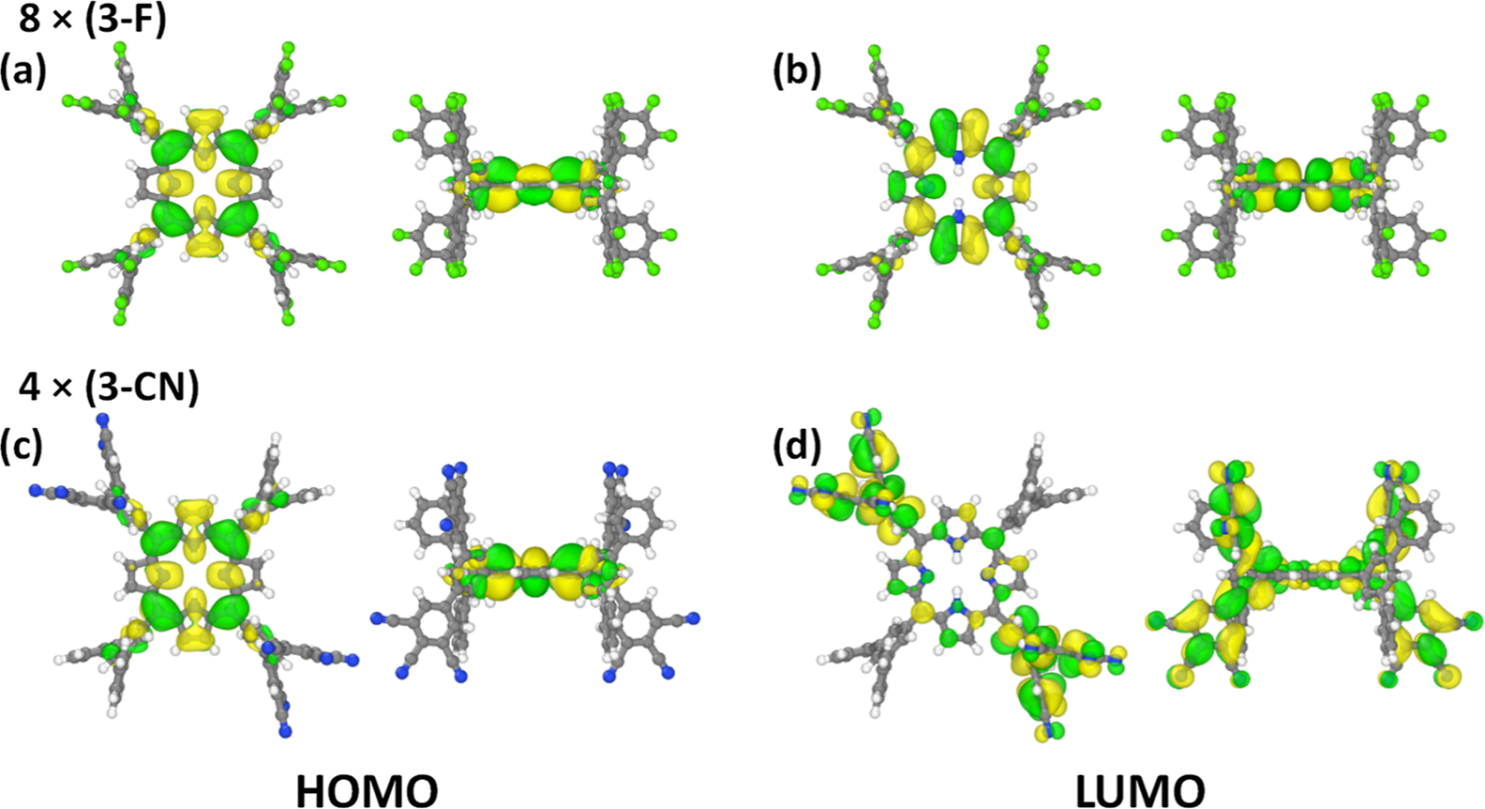
DFT-calculated isosurfaces illustrating the frontier orbitals (HOMO and LUMO) of 8 × (3 F) and 4 × (3-CN). Orbital plotted using Ovito 3.10.6.^43^ The isovalue has been set to ±0.012.

For the lowest unoccupied orbitals (LUMOs) the desired localization is also achieved for the F-substituted cases and in the fully –NH_3_-substituted system. It occurs to a lesser extent also for the fully –CN substituted molecule. In the partially –CN substituted cases, the effective symmetry of the LUMO is broken and the orbital gets increasingly localized on the peripheral, substituted rings due to the strongly electron withdrawing character of the –CN groups. In fact, when removing the bridging benzene, in the 1 × (3-CN)′, 2 × (3-CN)′, and 4 × (3-CN)′ systems the LUMOs are fully localized on the peripheral rings, while they appear largely localized on the central porphyrin for 6 × (3-CN)′ and 8 × (3-CN)′ (see Figure S2).

### 3.2 Electron Affinities and Ionization Energies

To assess the impact of the substituents on the electronic structure of the molecules, vertical ionization energies (IEs) and electron affinities (EAs) were calculated by subtracting the total energies of the respective ions from the neutral molecules. For the sake of comparison, the energies of the HOMOs and LUMOs of the neutral molecule were also analyzed as approximate quantities. Importantly, they follow the same trends as those discussed below for the IEs and EAs (see Figure S4).

For the fluorinated molecules, Figure 3a reveals an essentially linear increase of the ionization energies and electron affinities with the number of substituted rings. Interestingly, for the model systems lacking the bridging benzene rings exactly the same evolution is observed (minor absolute differences can be associated with the somewhat modified orbital localization). The equivalent trends support the notion that the observed shifts are generated by a “through space” effect caused by the change in electrostatic energy due to the highly polar substituted rings in the periphery of the molecules. This can be understood from the fact that the energies of the molecular eigenstates (which in a first approximation determine IE and EA) are defined relative to the local electrostatic energy. Thus, shifts of this energy reference relative to the vacuum level (i.e., the electrostatic energy at a nominally infinite distance from the considered molecule) causes concomitant changes of IE and EA.^44^ Keeping this in mind, the observation that the changes in IEs and EAs in Figure 3a are essentially the same for each system further supports the notion that the they have an electrostatic origin and are not caused by more specific, quantum-mechanical effects like orbital hybridization.

**Figure 3:**
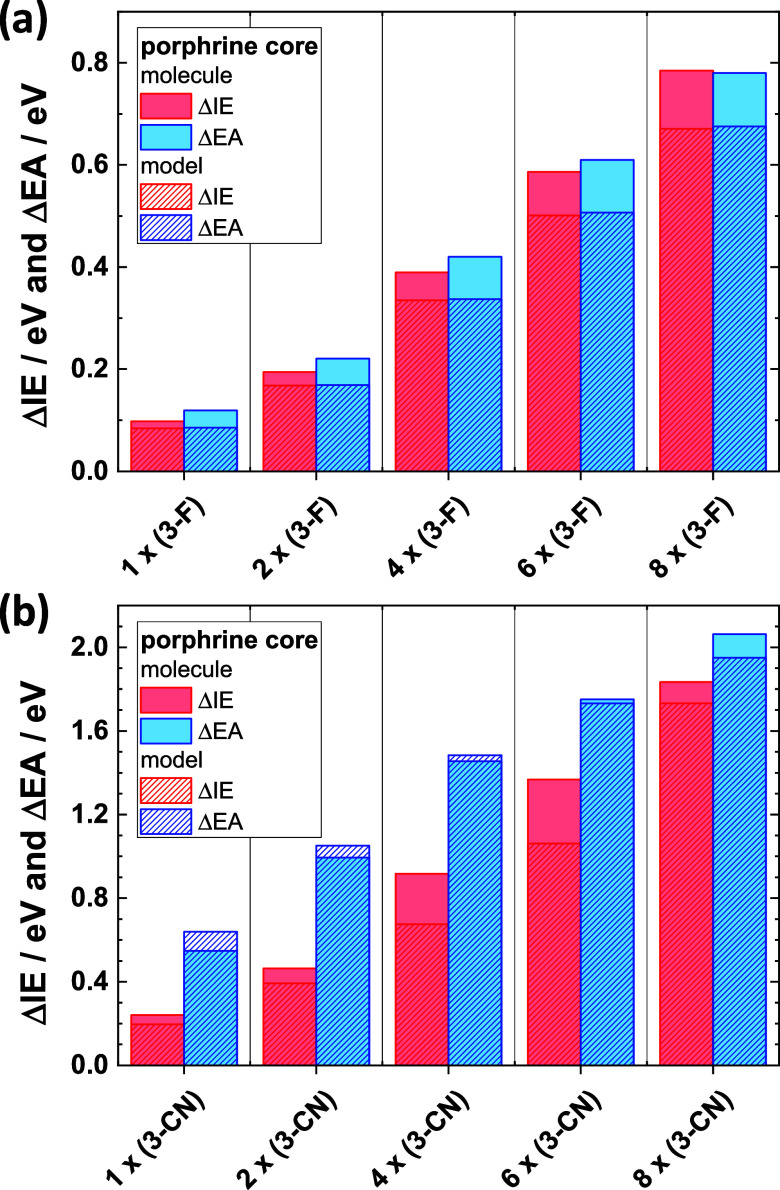
Change in vertical ionization energies, ΔIE, and electron affinities, ΔEA, upon increasing the number of triply –F (panel (a)) and –CN (panel (b)) substituted benzenes in the studied molecules (c.f., Figure 1). ΔIE and ΔEA are calculated as differences between the total electronic energies of ions and neutral molecules.

In the –CN substituted systems, the trend for the ionization energies is consistent with what has been observed for the fluorinated molecules. Only the magnitude of the increase in IE is significantly larger (with ΔIE = 0.78 eV and ΔIE = 1.83 eV for the fully –F and –CN substituted molecules, respectively). This can be rationalized by the much larger dipole moment of a benzene ring bearing three –CN groups (μ = 7.8 D) compared to one with three –F substituents (μ = 2.6 D). For the electron affinities, distinctly larger changes than for the IEs are calculated for the only partially substituted systems. According to what has been stated above, this appears inconsistent with an electrostatic (“through space”) origin of the shift. This conundrum can, however, be resolved by considering orbital localization: As discussed above, in the partially –CN substituted molecules, the unoccupied states are largely localized in the molecular periphery. There, “through bond” mesomeric and inductive effects cannot be neglected and, moreover, parts of the orbitals are particularly close to the polar groups such that also field-related effects are amplified.

An increase of IEs and EAs is consistent with the nature of the substituents, as can be inferred from Figure 4: inward-pointing dipoles causes an increase of the electrostatic energy (defined for positive charges) in the region of the porphyrin. This stabilizes all eigenstates of the (negatively charged) electrons such that it becomes energetically more costly to remove and more rewarding to add them.

**Figure 4:**
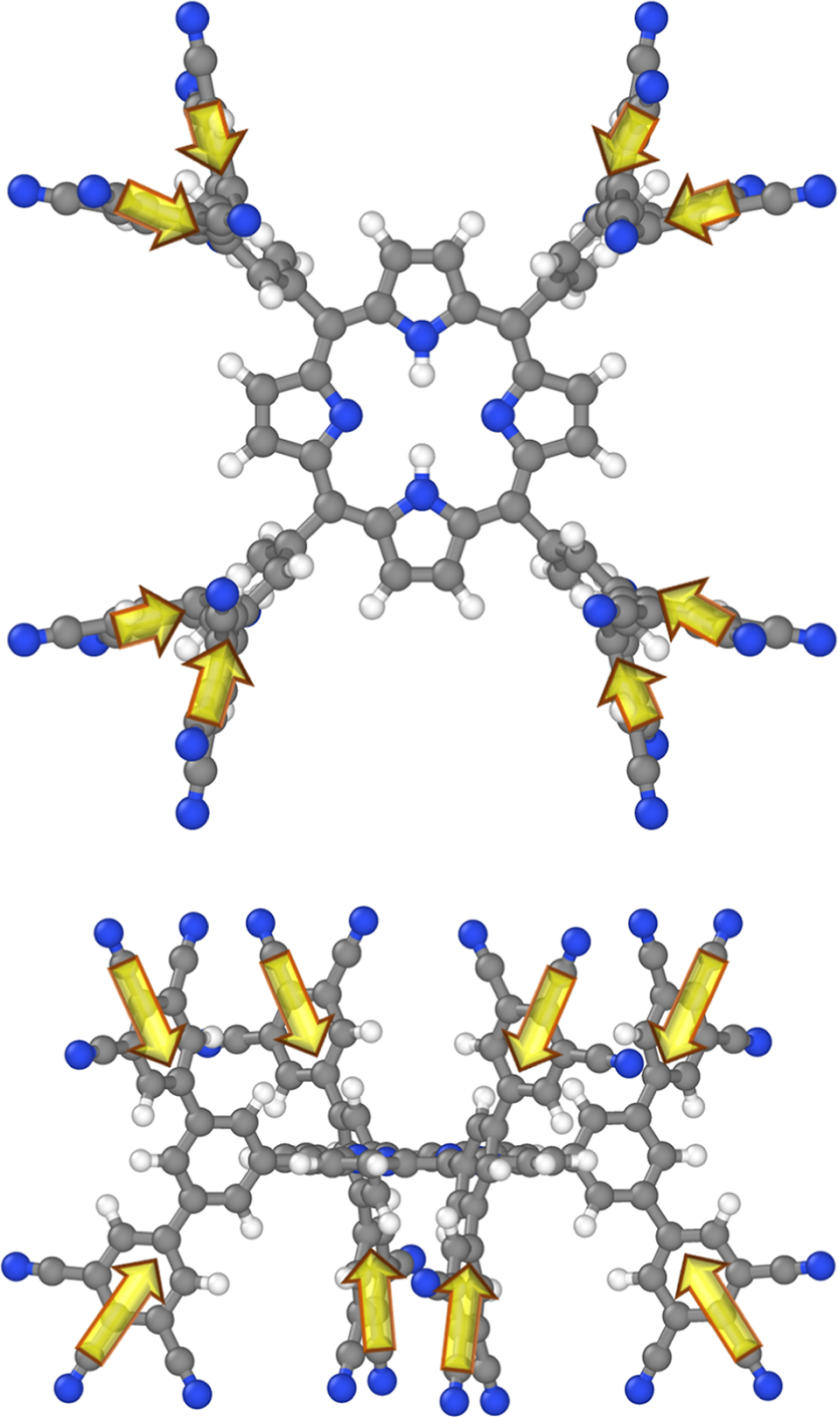
Schematic representation of the spatial dipole distribution around the porphyrin core for the molecule characterized by an 8 × (3-CN) substitution pattern (with all R-groups in Figure 1 substituted by –CN). An equivalent plot could be made for the –F substitution, while for –N­(CH_3_)_2_ substituents the arrows would point in the opposite directions. Note that, for the sake of simplicity, in this plot the complex charge distributions within the substituted benzenes are approximated by the first term of a corresponding Taylor expansion, i.e., by dipole moments (sketched as yellow arrows). Consistent with IUPAC conventions,^5^ dipoles point from the negative to the positive pole. Structure plotted using Ovito 3.10.6.^43^

When choosing electron donating instead of electron-accepting substituents, the direction of the dipoles is reversed. As a consequence, the ionization energies and electron affinities should decrease and this is exactly what happens in the simulations: for a fully dimethylamine-substituted system, the calculated shifts amount to −0.65 eV for the IE and −0.45 eV for the EA, respectively.

How do the above-discussed observations correlate with the picture of collective electrostatics outlined in the Introduction section? In collective electrostatics one typically encounters a situation in which individual dipoles have comparably little impact on the energetics of the system and only their (semi)­periodic arrangement on the surface of a geometrical object like a cube, a cylinder, or a plane result in a significant shift of electronic states.^15–21,24–26^ In contrast, in the molecules discussed here, even individual substituted benzenes have an impact. In this context, one, however, has to keep in mind that the idea of collective electrostatics has been developed for the “far field” case. I.e., it describes primarily the situation for objects at some distance from the polar layers. Even more importantly, as shown by Natan et al.,^18^ the distance at which one enters a “far-field electrostatic” situation is distinctly (by up to an order of magnitude) reduced for a densely packed monolayer of dipoles compared to an isolated dipole. This reduction, however, only occurs, when a sufficiently large number of dipoles are arranged.^18^ Thus, the question arises, whether one is rather in a “local-field electrostatic” situation, when directly attaching substituents to a molecule. To fully assess the situation for the substituted porphyrins studied here, it is useful to explicitly understand, how local shifts in electrostatic energy build up upon attaching an increasing number of polar substituents and to what extend the finally realized electrostatic energy distribution is consistent with the expectations from collective electrostatics. This will be discussed in the next section.

### 3.3 Electrostatic Energy

To illustrate the substitution-triggered buildup of the “pocket of electrostatic energy” in the region of the porphyrin core due to the polar groups, yet another set of model structures was generated that consists only of the peripheral, substituted rings. In this way, the purely electrostatic impact of these polar entities can be illustrated. Figure 5 shows the evolution of the electrostatic energy of an electron, Δ*E*_elstat‑e_, around the peripheral benzenes for varying degrees of substitution. This is done via isovalue plots for the fluorinated molecules and for the molecules bearing –CN substituents. The plots for the fully substituted systems are provided at the top of Figure 6 to avoid redundancies. Equivalent plots for reduced isovalues are found in Figures S5 and S6 in the Supporting Information.

**Figure 5:**
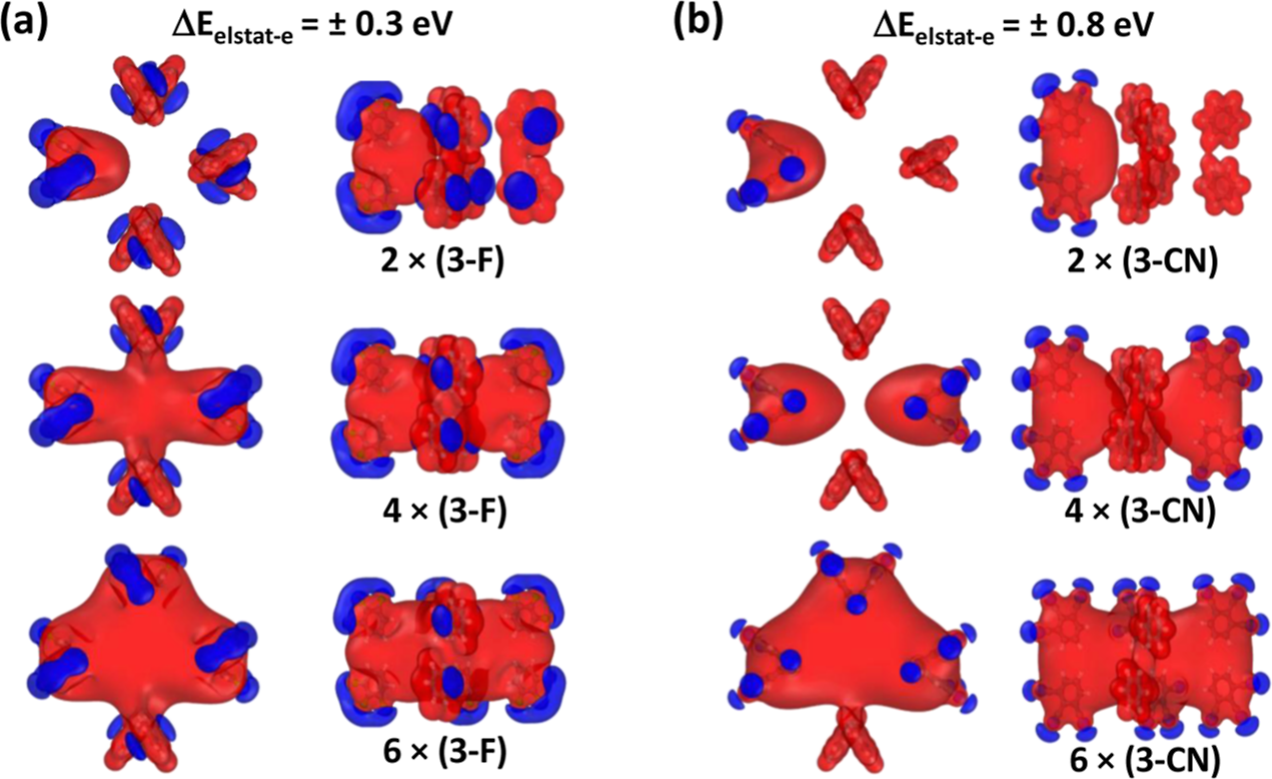
Change in electrostatic energy of an electron, Δ*E*_estat‑e_, due to the presence of eight benzene rings arranged at exactly the positions they adopt in the substituted porphyrins considered in this manuscript. The number of substituted (i.e., polar) benzene rings increases from the top (2 substituted rings) to the bottom (6 substituted rings). The plots for 8 substituted rings are contained in (Figure 6a,c). The energy changes are illustrated by means of isovalue plots connecting points at which the electrostatic energy changes reach the values specified on top of the plots. The red surfaces refer to decreases and the blue surfaces to increases in Δ*E*_estat‑e_. In the left part of the plot (panel (a)), the situation for the triply fluorinated benzenes is shown, while the right part of the plot illustrates the situation for –CN substituents (panel (b)). Isosurface and structures plotted using Ovito 3.10.6.^43^

**Figure 6:**
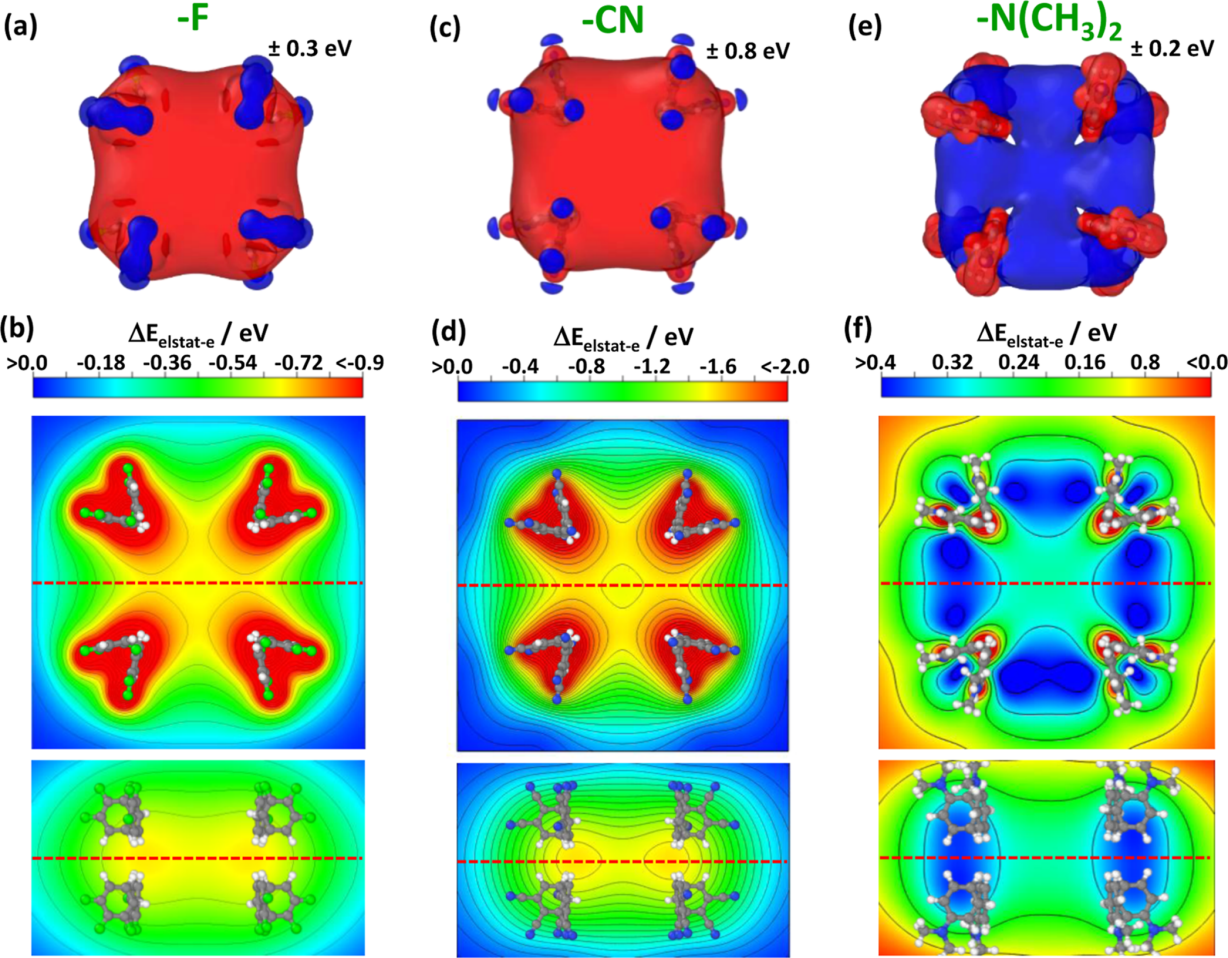
Change in electrostatic energy of an electron, Δ*E*_elstat‑e_ due to the presence of eight triply –F (panels (a,b)), eight triply –CN (panels (c,d)), or eight triply –N­(CH_3_)_2_ (panels (e,f)) substituted benzene rings. These are placed at exactly the positions at which they are found in the parent systems (the substituted porphyrins). In panels (a), (c), and (e), the energy changes are shown by means of isovalue plots as in Figure 5. In panels (b), (d), and (f), contour plots of the electrostatic energy in planes including the centers of the molecules with isolines drawn every 0.1 eV are provided. The red dashed line in the top-view indicates the plane for which the side view has been plotted and vice versa. Molecular structures are superimposed on the contour plots as guide to the eye. Both, the chosen isovalues as well as the color scales vary for the different materials. Isosurface and structures plotted using Ovito 3.10.6;^43^ energy cross sections plotted with VESTA 3.90.0a.^45^

In both substitution series, one clearly sees the impact of the Coulomb potential around the atoms forming the rings. For unsubstituted rings, this potential is, however, confined to the immediate vicinity of the rings and the change in electrostatic energy in the region, where usually the porphyrins would reside, is close to zero. This is different for substituted rings, where the changes in electrostatic energy reach further into space such that with an increasing number of polar benzenes a “pocket of shifted electrostatic energy” builds in the central region. I.e., one clearly sees that for molecular systems like the ones considered here, one is in a regime, in which even individual dipoles do have an impact, but where the overall shift in energy still builds up gradually with substituent numbers due to the superposition of the respective potentials.

As shown in Figure 6a,c,e, for fully substituted peripheral benzenes the pocket of electrostatic energy encompasses the entire region, where usually the porphyrin section of the molecules would be located. Notably, especially in that region the isosurfaces for the –CN and –F substituted rings look essentially the same, even though the isovalue for the –CN case is by *a* factor of nearly 2.7 larger. This testifies to the quantitatively significantly larger impact of the nitriles. The isovalue (and, therefore, the electrostatic shift) is smallest for the −N­(CH_3_)_2_ substituents and, most importantly, the sign of the shift is changed consistent with what has been discussed above for the IEs and EAs.

To obtain a more quantitative understanding of the situation, Figure 6b,d,e show the change in electrostatic energy in the plane in where the porphyrin would reside (top panels) and in a perpendicular plane comprising the center of the molecules (bottom panel). Again, qualitatively the situation for the F- and –CN substituted rings are the same, while quantitatively the effect is much larger in the latter case. For the –N­(CH_3_)_2_ substituents, not only the sign of the shift changes, but also the regions of maximum shift are not close to the substituted benzenes, but between them (blue regions in Figure 6f). This is primarily attributed to the fact that electrons experience a strongly attractive potential close to nuclei independent of potentially existing dipole moments. This amplifies the reduction of electron electrostatic energy close to the rings in panels (b) and (d), while it opposes the impact of the polar substituents in (f). The above observationis a clear indication that one is not yet in the far-field, “collective” regime but that the local atomistic structure still counts.

The difference between the local- and far-field situations (i.e., the cases of “collective” and “local” electrostatics) can also be demonstrated by a very simple model system: a cube on whose faces arrays of dipoles are arranged in a regular pattern. They do not directly reproduce the situation for the molecules discussed above, but they are still conceptually related and serve to illustrate the difference between collective and local effects. In Figure 7a,b the electrostatic energy of an electron is shown for 5 × 5 grids of inward-pointing dipoles on each face of the cube. Here, Δ*E*_elstat_ is essentially constant inside the cube (see yellow region in panel (a) and the region highlighted by the green square in panel (b)). Additionally, there is a distinct shift in the energy compared to the outside of the cube. Moreover, inside the cube the electrostatic energy hardly changes. This is the prototypical signature of collective electrostatics for extended (semi)­periodic arrangements of dipoles.

**Figure 7:**
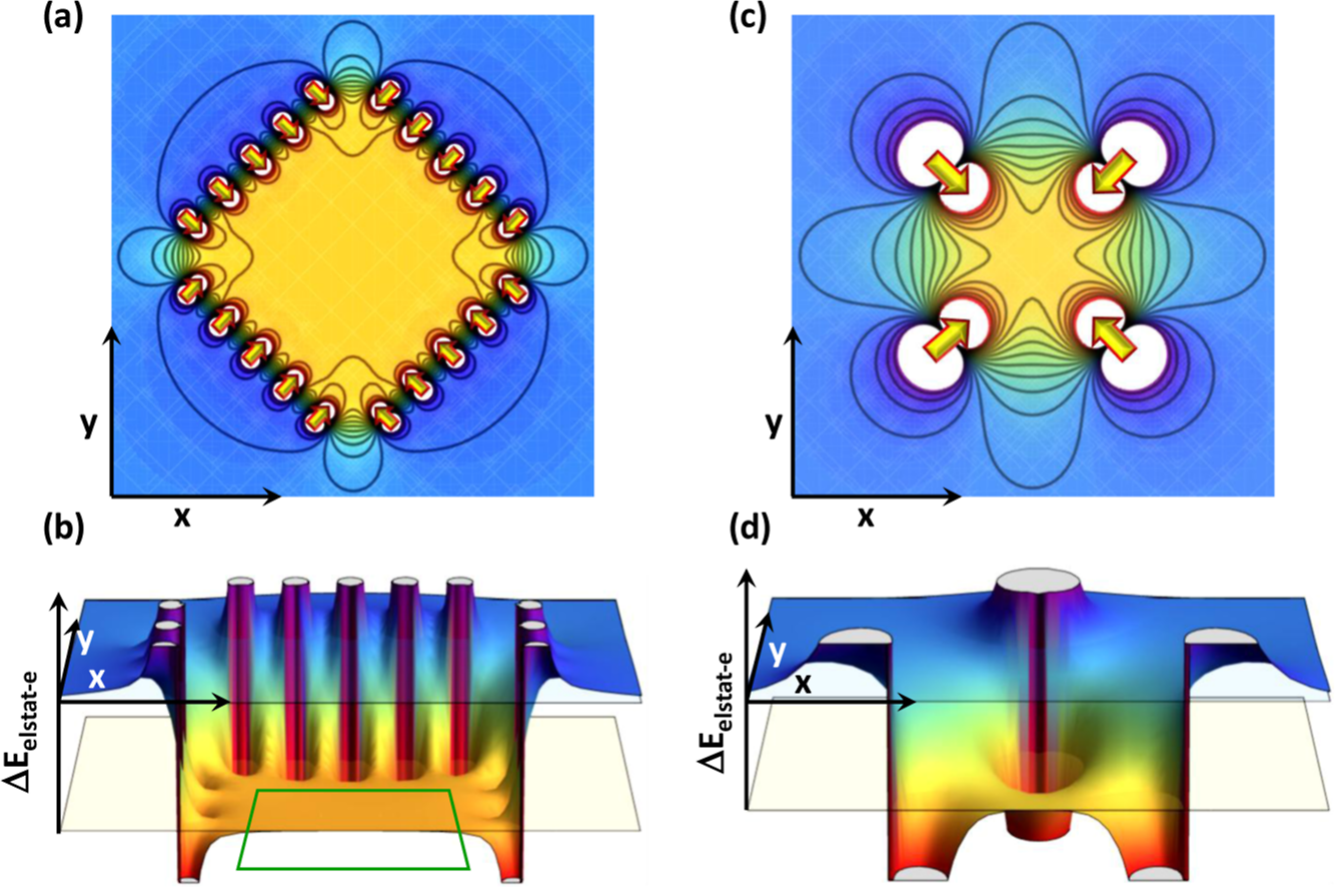
Change in electrostatic energy for cubic point dipole arrangements plotted for planes parallel to two faces of the cube and containing the center of the cube. Panels (a,b) show the situation for 25 dipoles per face of the cube arranged in a 5 × 5 grid, while panels (c,d) illustrate the case of only a single dipole per face. As the absolute values of the energy shifts depend on the geometric dimensions of the system and on the magnitudes of the dipole moments, which are not relevant for the conceptual considerations here, no values of the energy scale are specified. Panels (a) and (c) are contour plots similar to Figure 6b,d,f and are shown for the sake of comparability, while the energy is plotted as the vertical axis in a 3D plot in panels (b) and (d). The latter plots have been included, as they more clearly illustrate the plateau in the energy landscape inside the 5 × 5 cubes. All electrostatic energies were plotted using Mathematica 14.0 ^46^ based on the analytical expression for the electrostatic potential of point dipoles.

The situation is fundamentally changed, when each face of the cube contains only a single dipole (see panels (c) and (d)): Δ*E*_estat‑e_ still distinctly decreases in the center of the cube, but there is no longer an extended region of constant energy; rather one observes a pronounced decrease of the energy when approaching the dipoles and an increase between them. This is fully consistent with the situation shown in Figure 6b,d, and can be regarded as a classical example of local electrostatics.

### 3.4 Charge Rearrangements upon Ionization

To fully understand the correlation between the local electrostatic energy and the molecular IPs and EAs, it is useful to understand, in which region the charge is added or removed in the course of an ionization process. So far, this has been roughly estimated based on the shapes and localizations of the frontier orbitals (see above). This is, however, only a crude approximation, as one can expect significant electronic polarization processes following ionization events. This is illustrated in Figure 8a, where one sees that the largest rearrangements occur on the porphyrins and the linking benzenes, but that charge rearrangements on the peripheral substituted benzenes are non-negligible. This is insofar interesting as the peripheral rings comprise the majority of the heavy atoms of the molecules (two-thirds in the case of the fully –CN substituted system). Thus, to assess to what extent these charge rearrangements are consistent with (largely) localized net charges due to ionization processes, changes in atomic charges were calculated choosing the Hirschfeld partitioning scheme.^47^ As atomic charges are no observables, their calculation will always be approximative. Nevertheless, adding up atomic Hirshfeld charges in specific regions of the molecules reveal certain trends: upon electron removal, the majority (∼72%) of the positive net charge resides on the porphyrin and the bridging benzenes; the same applies to electron addition in the 8 × (3 F) molecule, while in the 8 × (3-CN) case only 43% of the extra negative charge is localized in that central region of the molecule. At first glance, this difference in the net charges might seem somewhat surprising, as for the 8 × (3-CN) the charge rearrangement on the outer rings seems to be only somewhat larger for electron addition than for electron removal. In this context, one, however, has to keep in mind that in the plots in Figure 8a net charging is superimposed with local polarization. Moreover, regarding the shapes of the charge rearrangements, one has to keep in mind that any change in the π charge-density, due to the orthonormality of the wave functions, will also result in a charge-redistribution in the σ-states.

**Figure 8:**
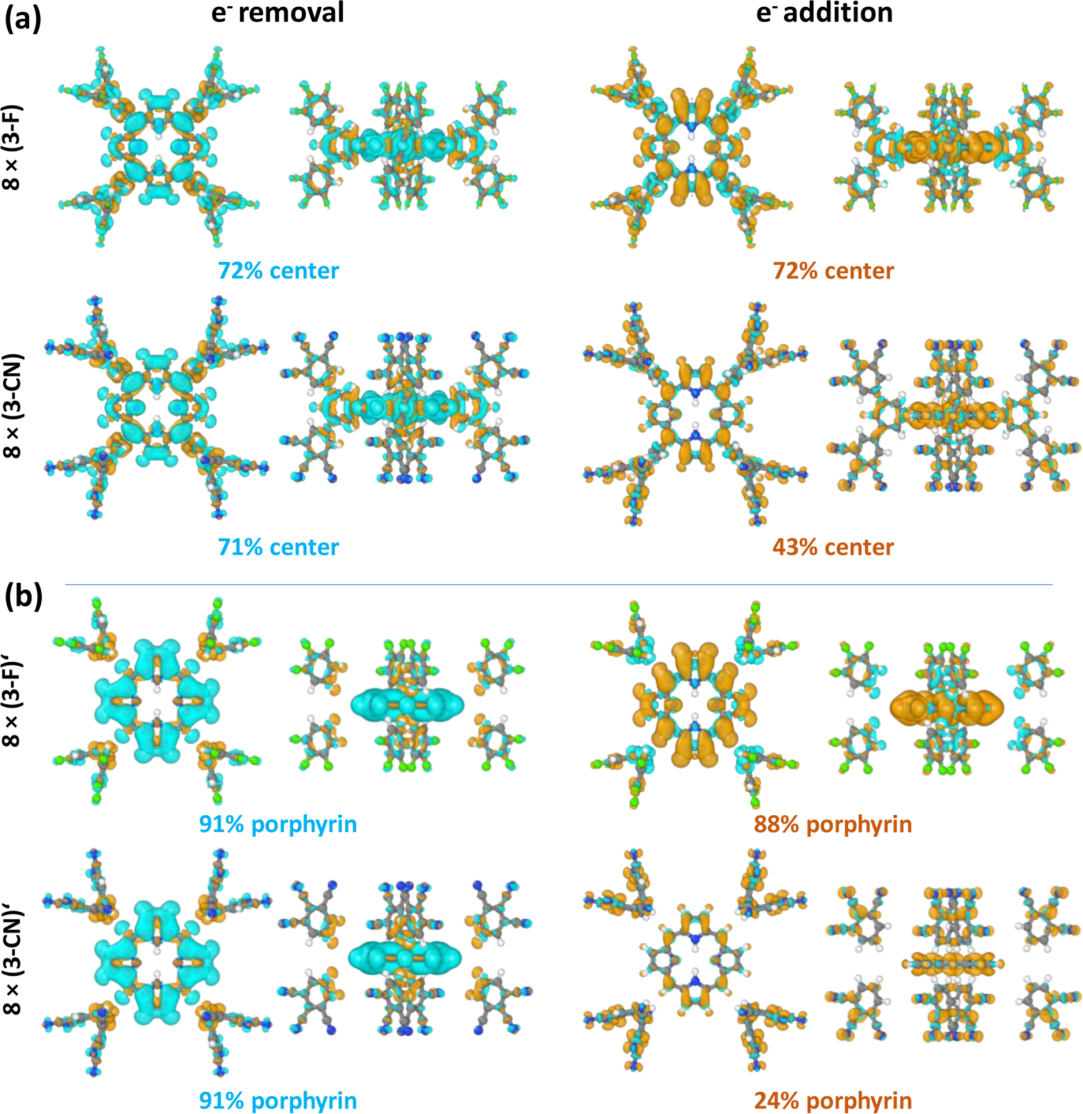
Charge rearrangements upon cation formation (left plots; associated with the ionization energies) and anion formation (right plots; associated with the electron affinities) for the fully fluorinated molecule (8 × (3 F); top row of panel (a)), the fully –CN substituted one (8 × (3-CN); bottom row of panel (a)) and for the respective model systems lacking the bridging benzenes (8 × (3 F)′ and 8 × (3-CN)′; panel (b)). The specified percentage values represent the percentage of charge removed from/added to the porphyrin (and in panel (a) the bridging benzenes) as calculated by adding up Hirshfeld^47^ atomic charges. The charge rearrangements have been determined for vertical ionization processes and have been calculated by subtracting the charge densities of the ionized and neutral molecules. Cyan isosurfaces correspond to positive charge densities and orange ones to negative charge densities (isovalue: 4·10^–4^ atomic units).

Overall, the charge rearrangement data show that the net changes in electron density yield localization trends that are largely consistent with those inferred from the shapes of the frontier orbitals (see Section 3.1), although the localization of the electron additions/removals appears less complete. The resulting observation that the majority of the added electron density for 8 × (3-CN) resides in the periphery of the molecule is consistent with the observation that for the –CN substituted molecules ΔEA, is always larger than ΔIE (see Figure 3). The peculiar situation for negatively charging the CN-substituted molecules becomes even more apparent, when considering the situation of the model system lacking the bridging benzene rings, as shown in Figure 8b: for electron removal and for electron addition in 8 × (3 F)′ the charging of the systems is essentially restricted to the central porphyrins and the peripheral rings primarily experience polarization effects. In contrast, for negatively charging 8 × (3-CN)′ the majority of the extra charge is localized on the now isolated peripheral rings, whose electronic states have been massively stabilized by the –CN substituents. In passing, we note that the equal charging of all peripheral rings appears as an artifact of the used semilocal functionals (which, however, does not pose a serious problem considering the purpose of the design of the model systems). Technically, the aforementioned situation is, in fact, reminiscent of the conceptually similar situation of integer charge-transfer processes at weakly coupled metal–organic interfaces. There, a careful tuning of the amount of exact exchange had been necessary to correctly describe the coexistence of charged and neutral adsorbate molecules upon interfacial charge transfer.^48^

Overall, the discussion of the charging-induced charge rearrangements shows that qualitatively the earlier arguments relying on orbital shapes prevail, but that for a full understanding of the ionization process of the molecules (including local polarization effects) it is useful to consider their actual charging.

## 4 Conclusion

In the current manuscript, density-functional theory calculations are used to illustrate the massive change in ionization energies caused by through-space interactions due to polar molecular building blocks. The underlying changes in the electronic structure are a direct consequence of the electrostatic potential arising from the superposition of the individual potentials of polar entities arranged in the molecular periphery. This is demonstrated for a model system consisting of a porphyrin core substituted by four bridging benzenes. To the latter eight additional benzenes are attached. These at least in part bear strongly accepting or donating substituents. Through-bond interactions are eliminated by a strong twist between the planes of the individual conjugated segments and by linking them in meta-position. In most cases, this also significantly localizes the frontier states on the central porphyrins. As a consequence of the polar substituents, shifts of IEs and EAs of up to ∼2.0 eV are predicted with the maximum value found for a heavily nitrile-substituted molecules. As all simulations were performed in the gas phase, it should be mentioned that due to its purely electrostatic origin, the calculated shifts can potentially be reduced by polarization effects, when the molecules are surrounded by highly polar media. In contrast, polarization effects within the molecules, which upon electron addition or removal extend over the entire molecular structure, are fully accounted for.

Considering the electrostatic nature of the observed changes in the molecular electronic structure, one is reminded of collective electrostatic effects intensively discussed in the past decades especially for interfaces and surfaces comprising regular (semi)­periodic arrangements of dipoles.^15–21,24–26^ These effects, for example, determine the interfacial energy-level alignment and can change contact resistances in hybrid devices by orders of magnitude.^49,50^ A closer inspection, however, reveals that there are several distinct differences between collectively induced shifts in energy levels and the observations at the molecular level: individual polar entities (or individual missing dipoles in periodic assemblies)^21,25^ have hardly any impact in the realm of collective electrostatic. In contrast, for the molecules studied here already individual substituted benzenes in the molecular periphery change IEs and EAs. Overall, the evolution of these quantities with the number of substituted rings is essentially linear. Moreover, in collective electrostatics the periodically arranged dipole sheets always generate extended regions in space, in which the electrostatic energy is strongly shifted but does not vary locally.^18^ Such extended regions of essentially constant electrostatic energy are not observed in the molecules studied here. This illustrates that at the molecular level the conditions for collective electrostatics are not fully fulfilled at least in the system studied here: (i) the π-conjugated porphyrin core is effectively too close to the polar groups, especially as (ii) even eight highly polar rings in the molecular periphery do not fulfill the condition of a (semi)­periodic arrangement of dipoles. In fact, as shown in the seminal paper by Natan et al.,^18^ both reasons are tightly connected, as a regular assembly of polar groups would significantly reduce the real-space distance at which the transition between the “near-field” and the “far-field” situation occurs. In that sense, the present situation, which is determined by the superposition of the fields of multiple dipoles attached to a single molecule, could be characterized as a “cumulative local-field case”.

As a consequence, electrostatic through-space interactions can have a massive impact on a molecule’s electronic structure, but they follow rules that are in various aspects different from the electrostatic effects commonly observed at interfaces.

## Supplementary Material



## Data Availability

All relevant input and output files concerning the data shown in the current manuscript are publicly available at the TU Graz data repository: DOI 10.3217/j24cj-qem24.

## Notes

Inner-sphere relaxation energies for various molecular systems calculated with different basis sets; graphical visualization of the frontier orbitals of all considered systems; approximate dipole-induced shifts of IEs and EAs inferred from orbital energies; isovalue plots of the electrostatic energies of all studied systems for different sets of isovalues (PDF)
