# Supplementary material for: Collective Electrostatics vs through-Space Interactions: Electronic Properties of Molecules with Multiple Polar Substituents
Source: ACS Phys Chem Au. 2025 Nov 24;6(1):174–84. doi: 10.1021/acsphyschemau.5c00104 (PMC12856644; doi:10.1021/acsphyschemau.5c00104)
Supplement: Supplementary file 1 [file pg5c00104_si_001.pdf]

Supporting Information for

Collective electrostatics vs. through-space interactions: electronic properties of molecules with multiple polar substituents.

*Egbert Zojer**

Institute of Solid State Physics, NAWI Graz, Petersgasse 16, A-8010 Graz, Austria

E-mail: Egbert.zojer@tugraz.at

Table S1: inner-sphere relaxation energies (in meV) for cation- and anion-formation (λ^+ and λ^-) for porphyrin, for the unsubstituted molecule from Figure 1 in the main manuscript and for 8 × (3-F) as well as 8 × (3-CN). When two basis sets are specified, the first one refers to the one used in the geometry optimizations of neutral and charged molecules and the second one describes that for single-point calculations performed to determine total energies; if only one basis set is specified, it was used for both purposes.

	λ^+	λ^-	λ^+	λ^+	λ^+	λ^+
Basis set	6-31G(d,p)	6-31G(d,p)	6-31G(d,p)/ 6-311+G(d,p)	6-31G(d,p)/ 6-311+G(d,p)	6-311+G(d,p)	6-311+G(d,p)
porphyrin	96 meV	152 meV	150 meV	97 meV	102 meV	146 meV
parent molecule	65 meV	103 meV	37 meV	65 meV		
8 × (3-F)	51 meV	109 meV	34 meV	71 meV		
8 × (3-CN)	61 meV	37 meV	32 meV	9 meV		

The inner-sphere relaxation energies calculated as differences between total energies of cations or anions obtained for neutral and charged equilibrium geometries. They are extremely small in all cases. Thus, it is not overly surprising that variations in the basis set cause changes in the numerical values that for the more extended systems are non-negligible on a relative scale (c.f., errors of differences of large numbers). Still, the variations are very small on an absolute scale.

Independent of the used methodology one observes that:

- All relaxation energies are exceptionally small
- In the extended systems the relaxation energies are smaller than in porphyrin, which can be rationalized by the more delocalized distribution of the excess charge
- The relaxation energy of the parent system and the substitute molecules for a specific charge or basis set are very similar, such that one can conclude that the substituent effect is virtually identical for vertical and adiabatic quantities (with the exception of the peculiar case of the anion of 8 × (3-CN) – see also main text)

LUMO 8 × (3-F)

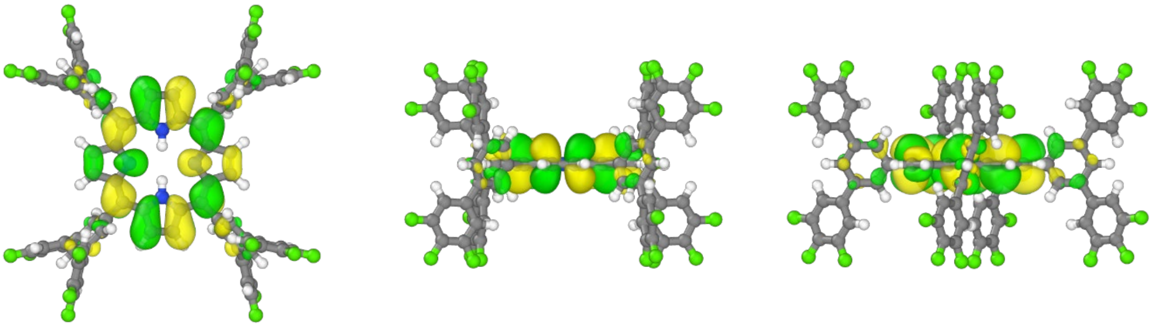

HOMO 8 × (3-F)

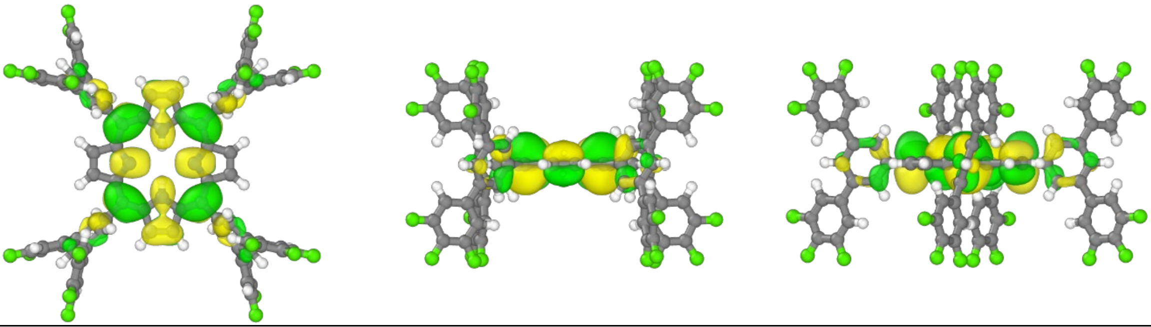

LUMO 6 × (3-F)

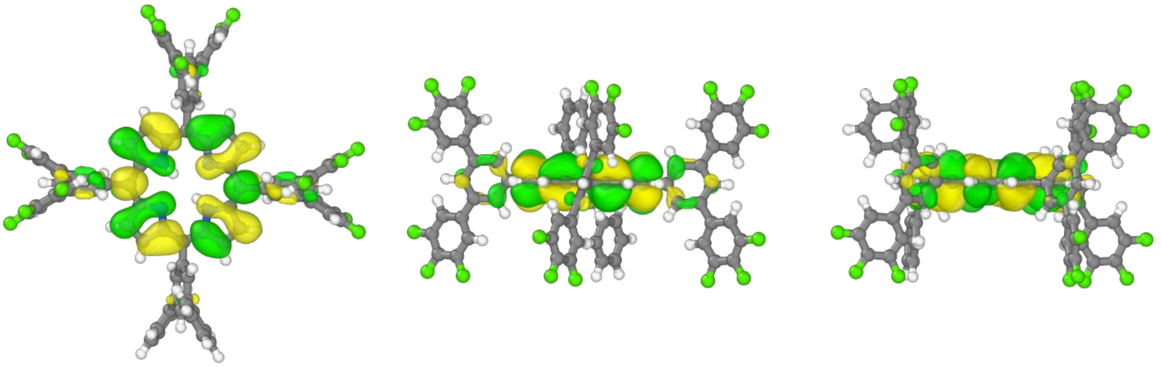

HOMO 6 × (3-F)

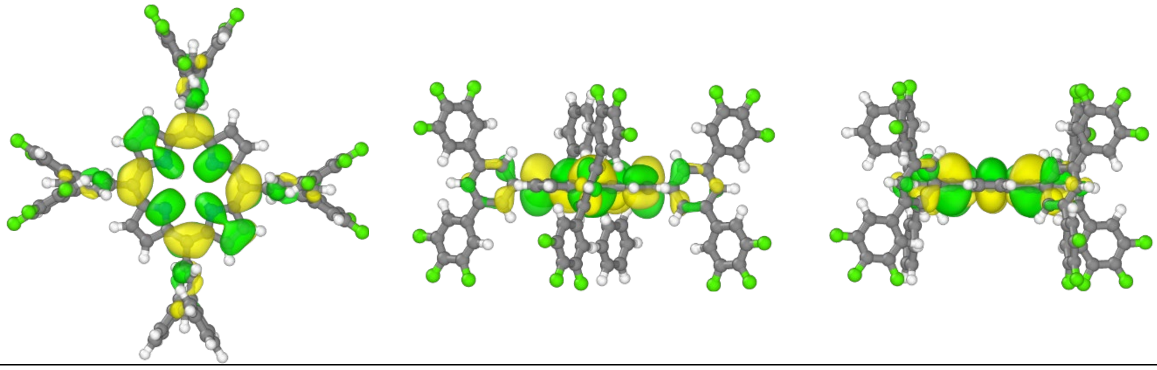

LUMO 4 × (3-F)

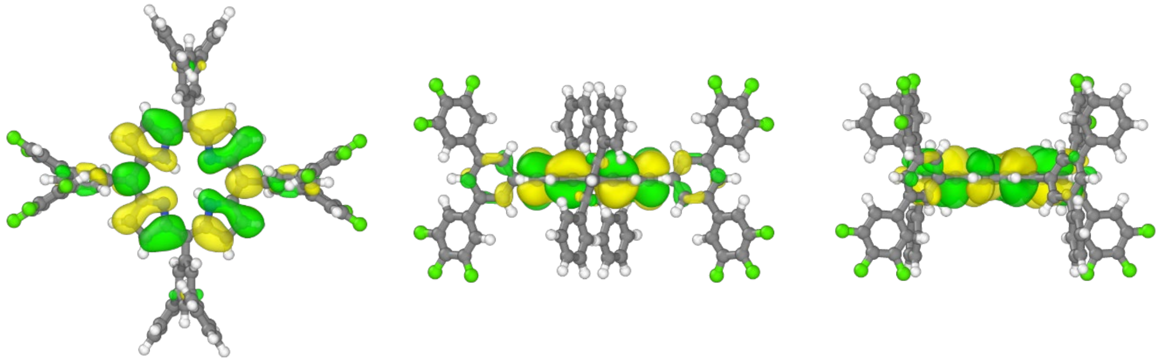

HOMO 4 × (3-F)

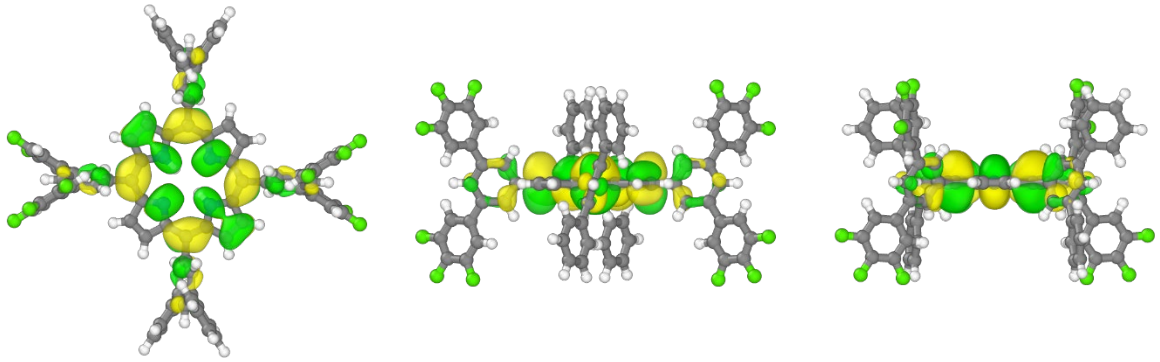

LUMO 2 × (3-F)

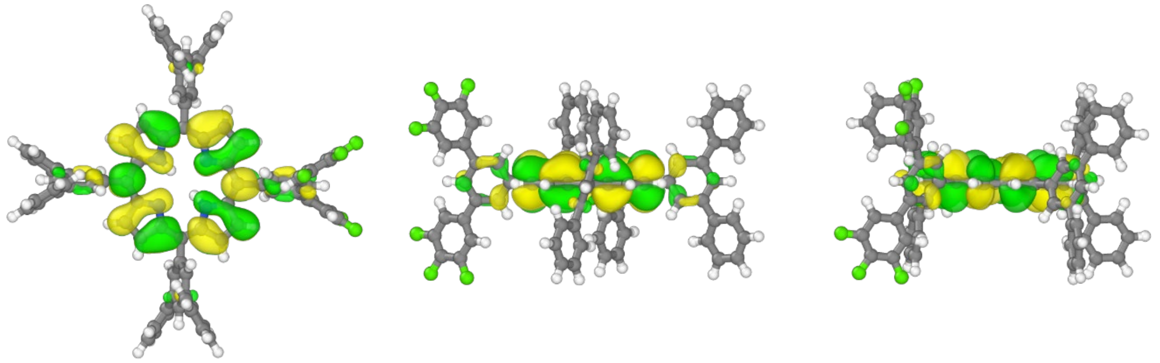

HOMO 2 × (3-F)

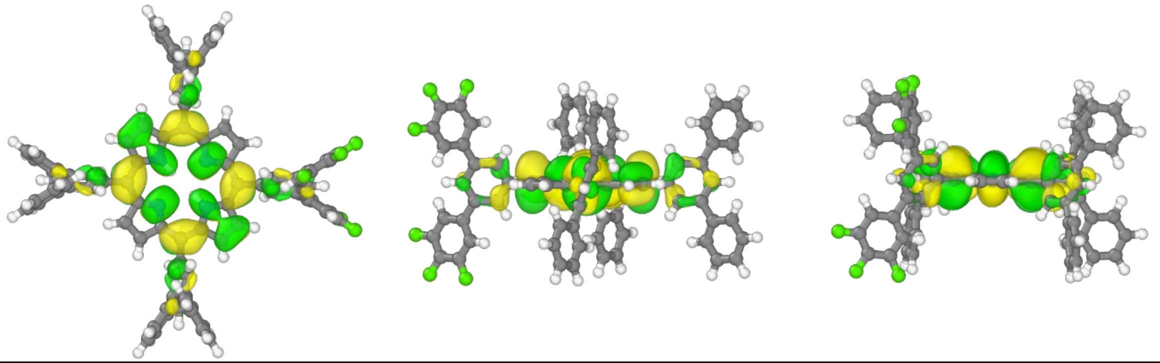

LUMO 1 × (3-F)

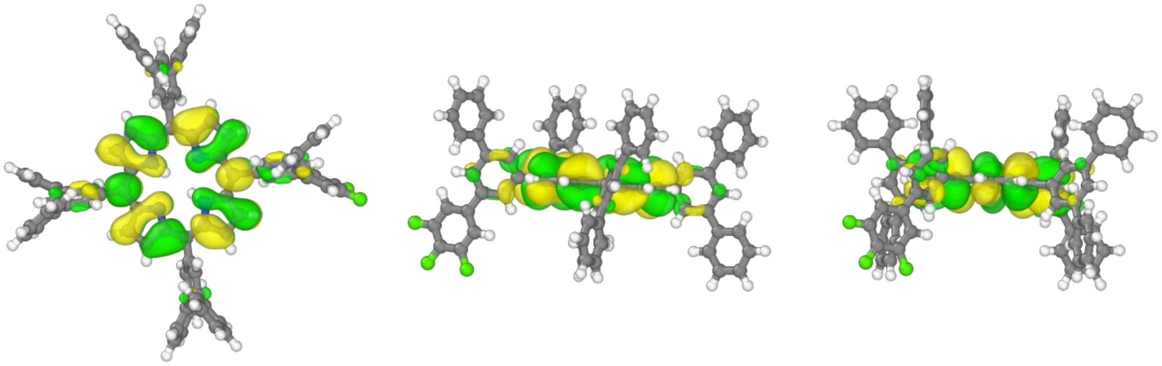

HOMO 1 × (3-F)

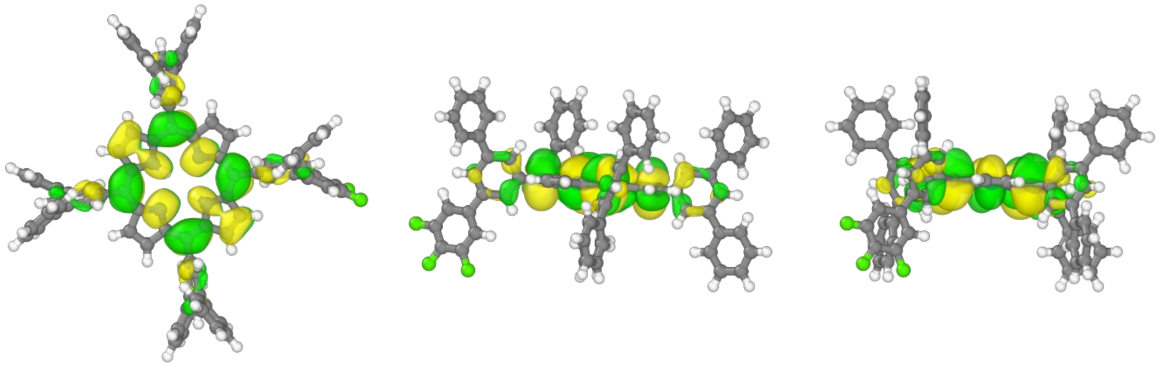

Figure S1: Isosurfaces illustrating the frontier orbitals for the F-substituted systems for a decreasing degree of substitution (from top to bottom)

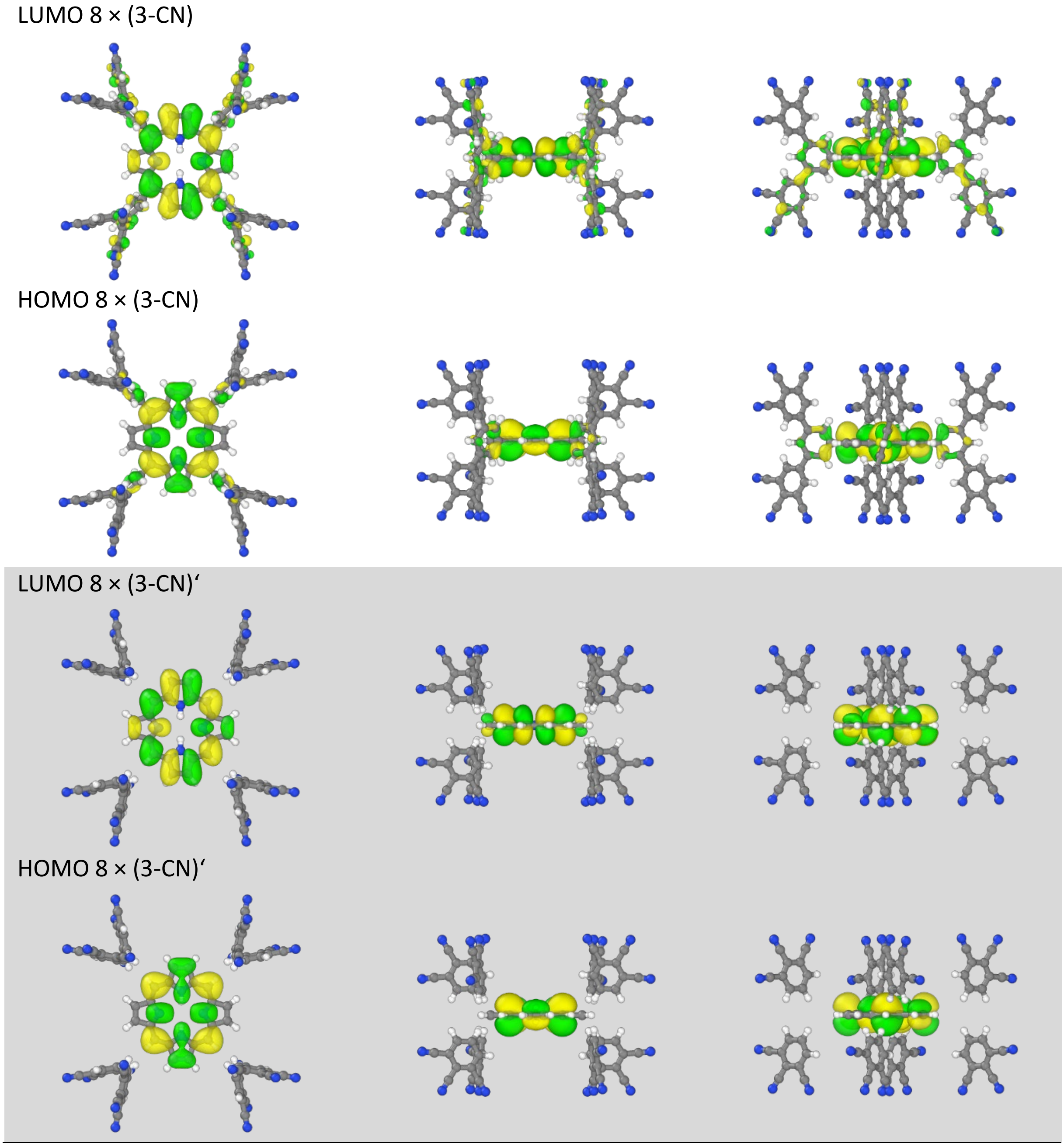
LUMO 8 × (3-CN)
HOMO 8 × (3-CN)
LUMO 8 × (3-CN)‘
HOMO 8 × (3-CN)‘

LUMO 6 × (3-CN)

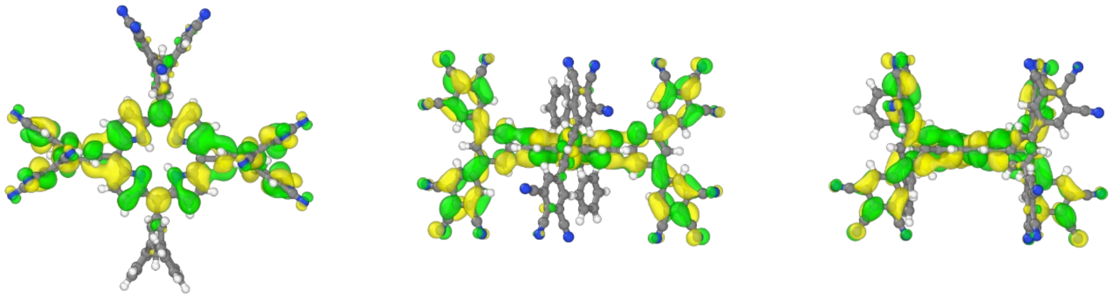

HOMO 6 × (3-CN)

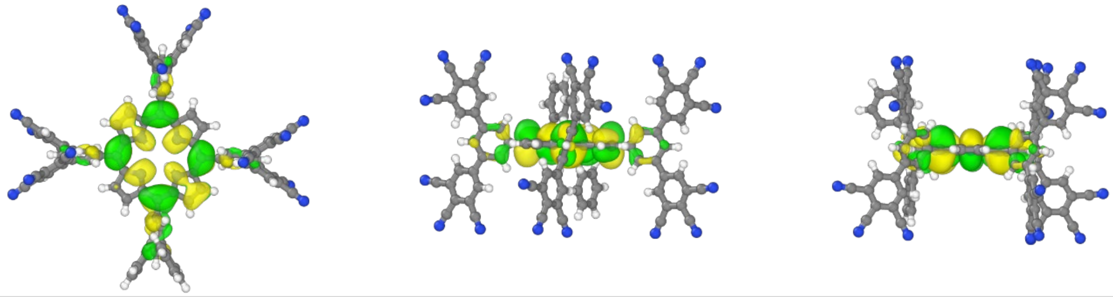

LUMO 6 × (3-CN)‘

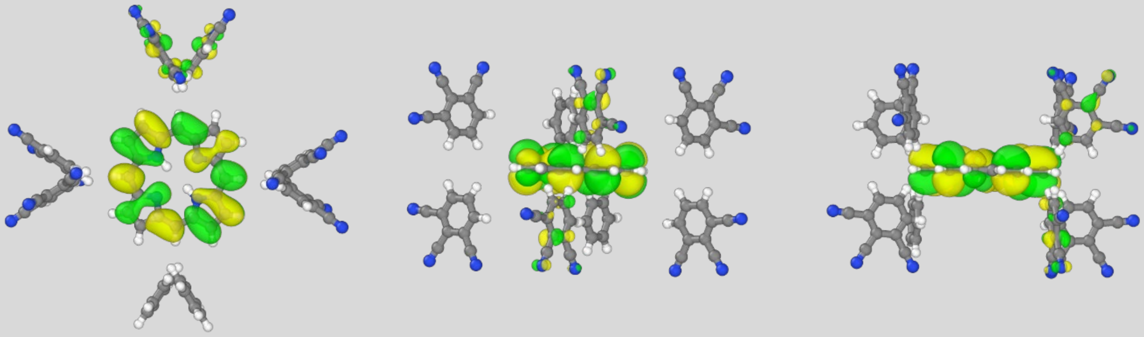

HOMO 6 × (3-CN)‘

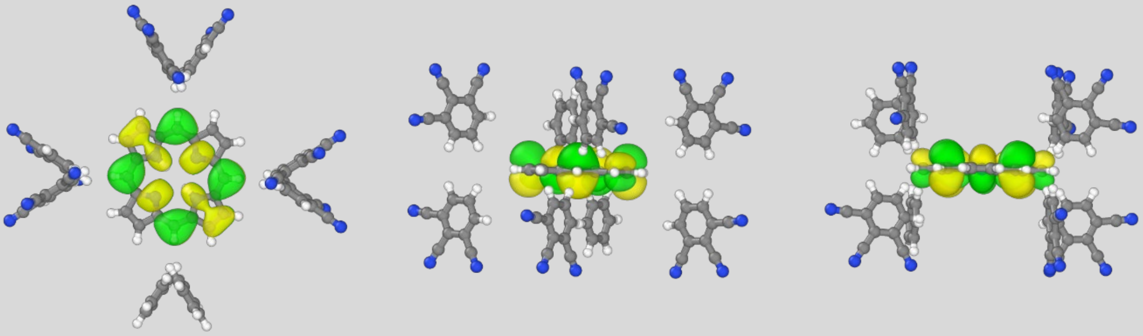

LUMO 4 × (3-CN)

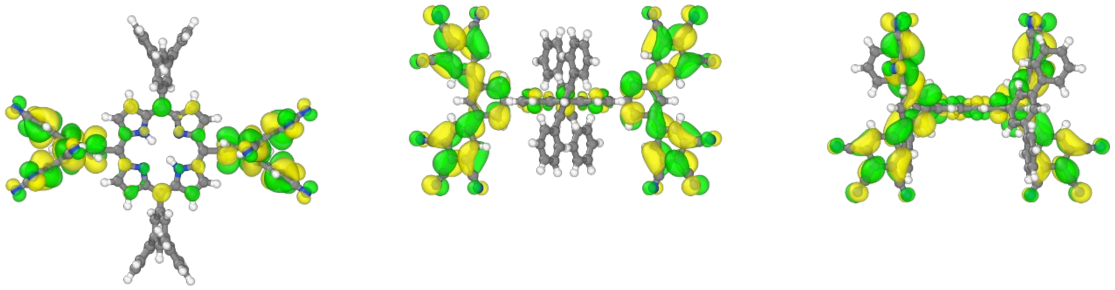

HOMO 4 × (3-CN)

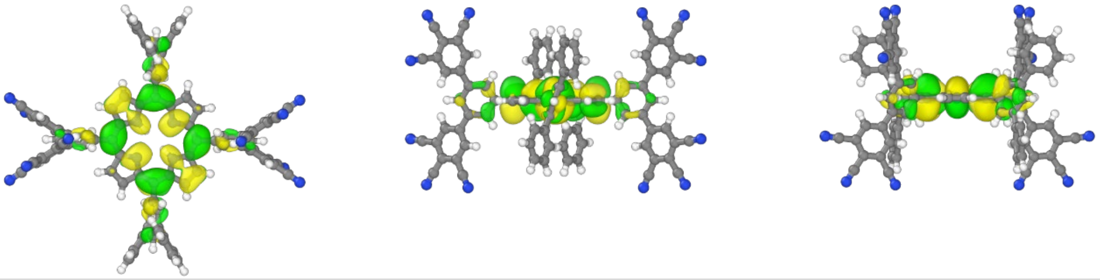

LUMO 4 × (3-CN)‘

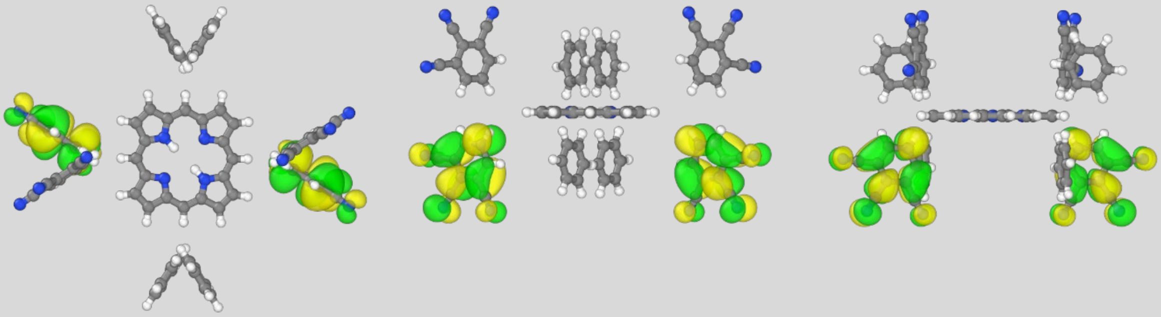

HOMO 8 × (3-CN)‘

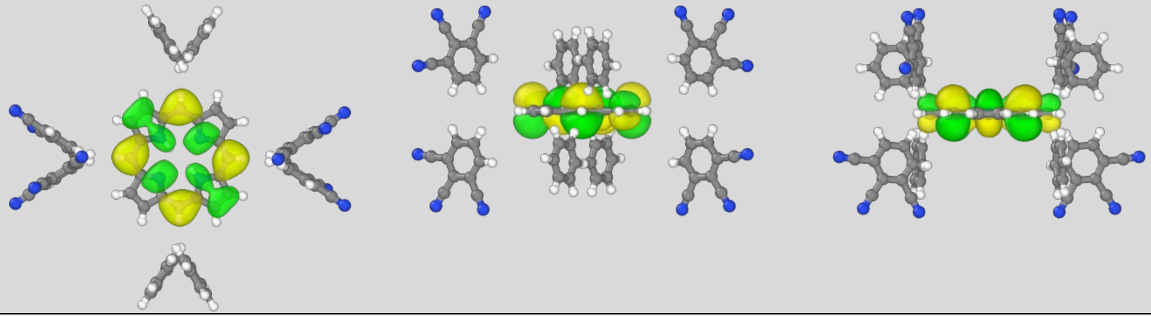

LUMO 2 × (3-CN)

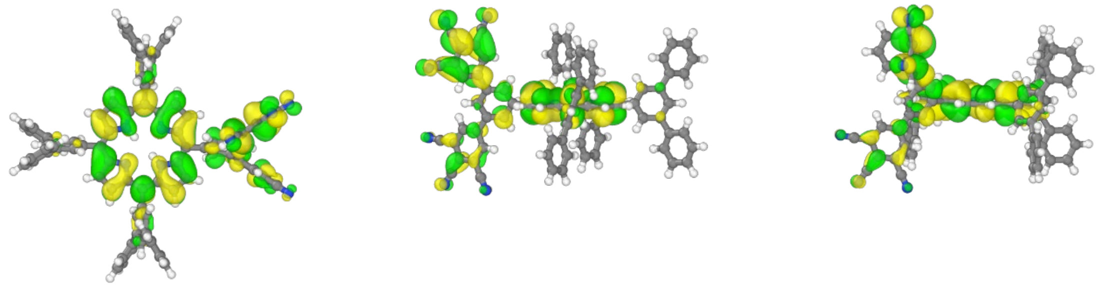

HOMO 2 × (3-CN)

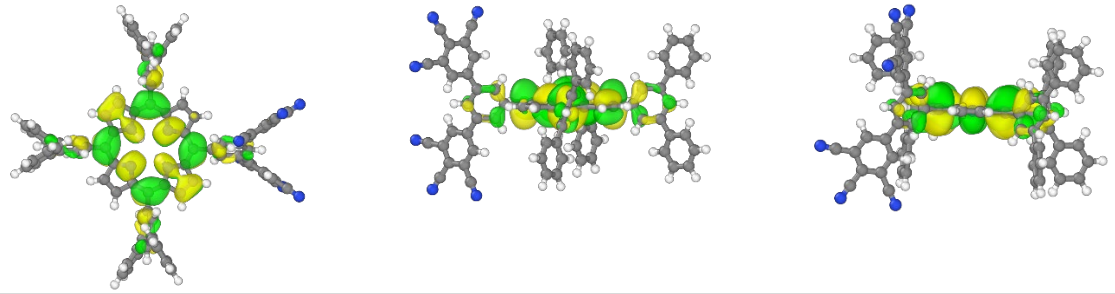

LUMO 2 × (3-CN)‘

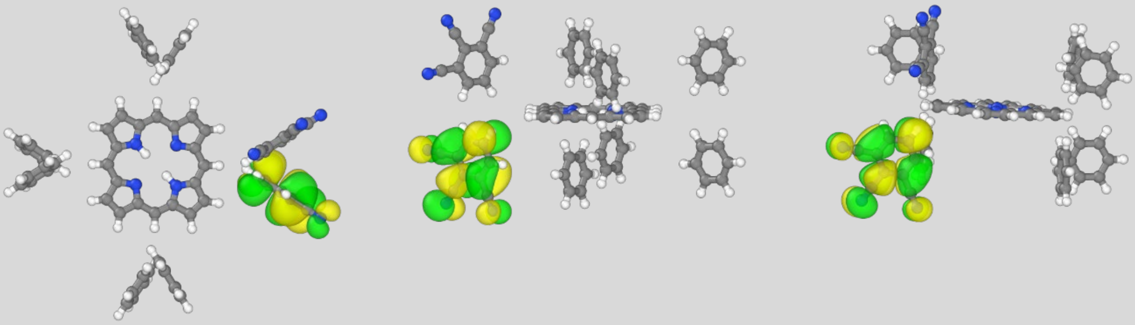

HOMO 2 × (3-CN)‘

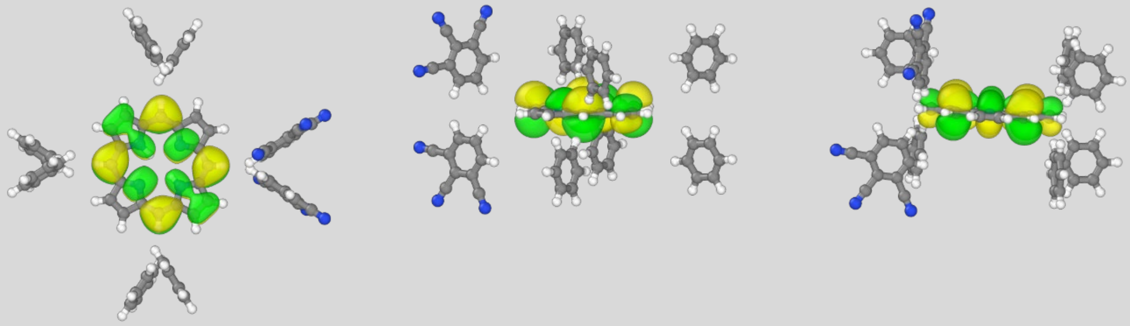

LUMO 1 × (3-CN)

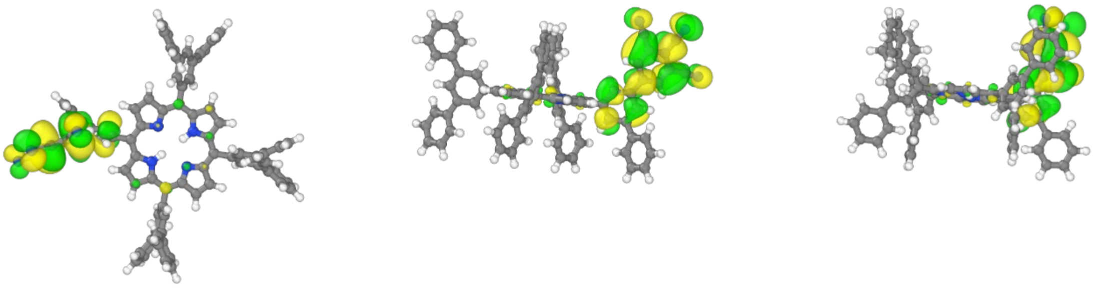

HOMO 1 × (3-CN)

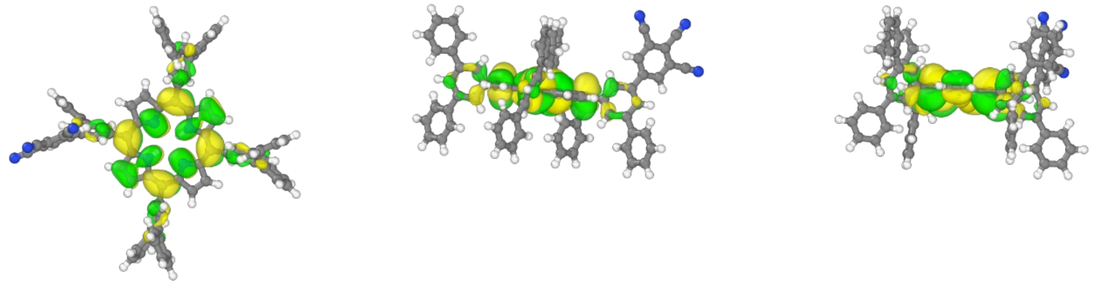

Figure S2: Isosurfaces illustrating the frontier orbitals for the CN-substituted systems for a decreasing degree of substitution (from top to bottom); plots with a grey background show the situation for model systems missing the intermediate benzene rings.

LUMO 8 × (3- $N(CH_3)_2$)

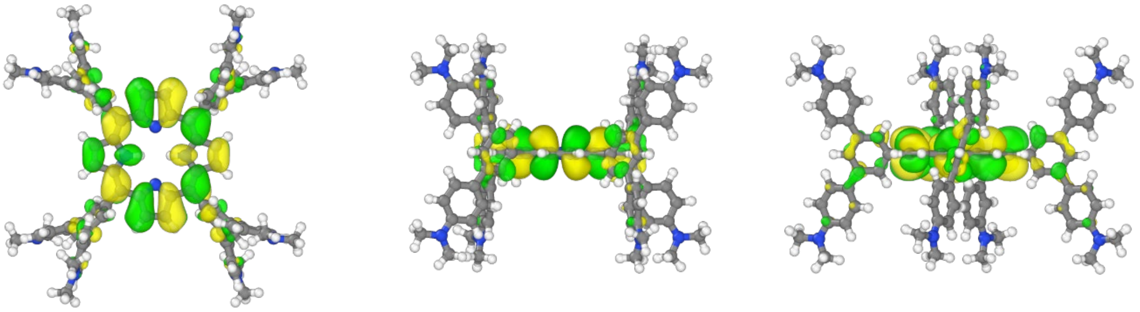

HOMO 8 × (3- $N(CH_3)_2$)

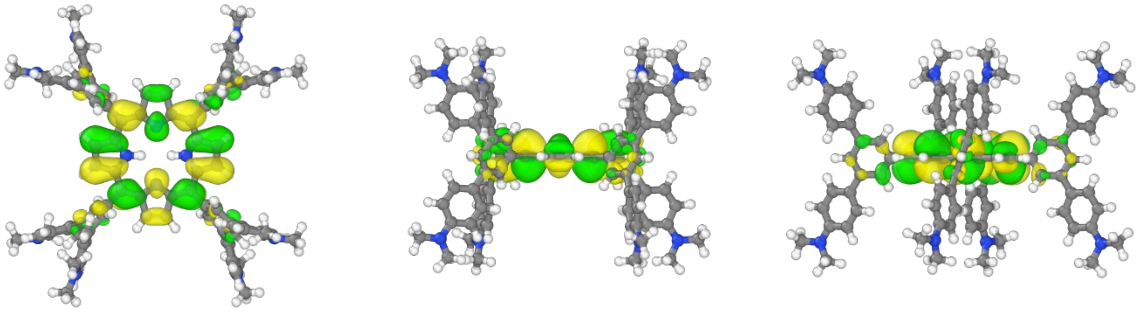

Figure S3: Isosurfaces illustrating the frontier orbitals for the fully $N(CH_3)_2$-substituted systems

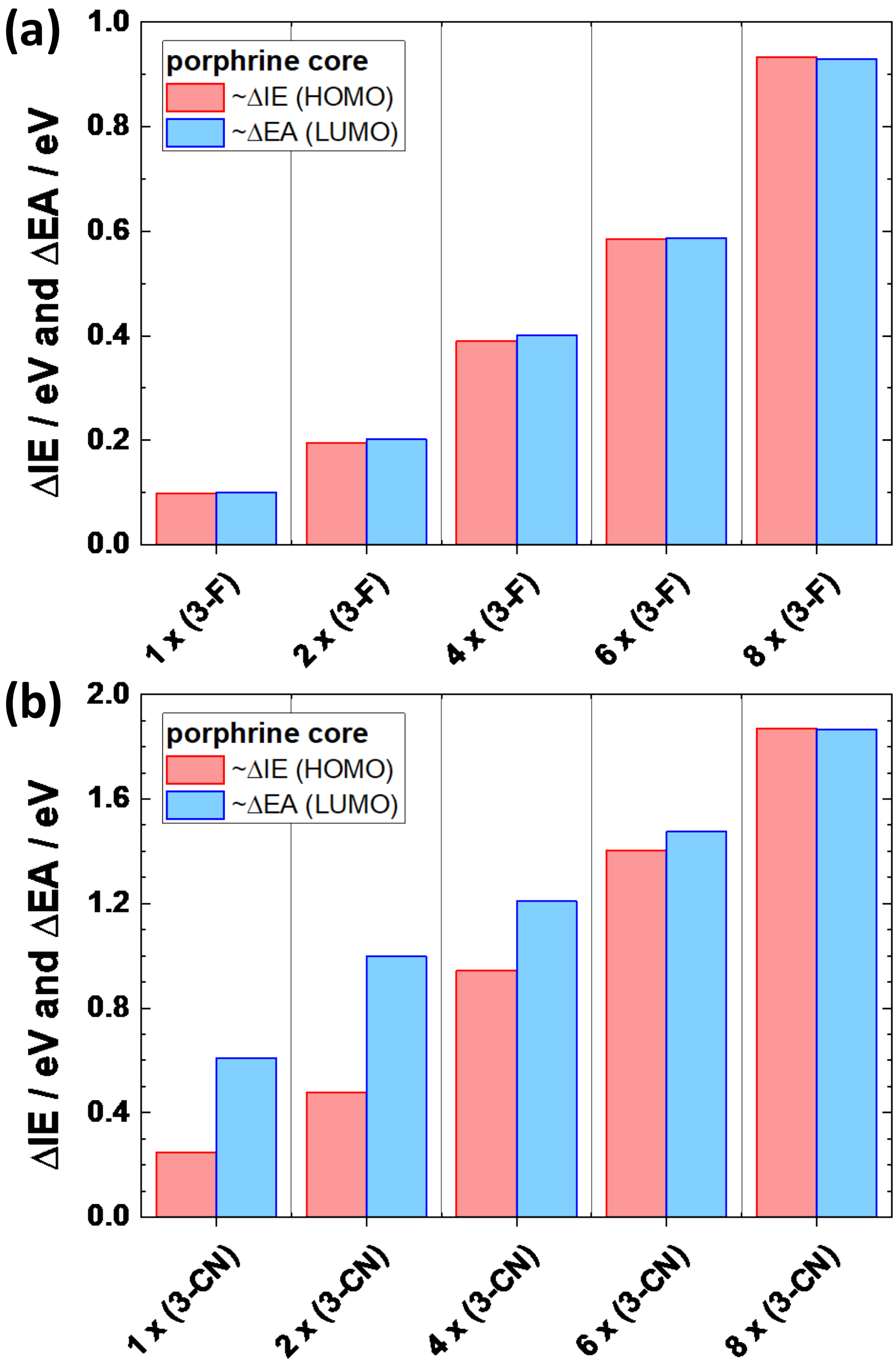

Figure S4: Approximate values for the change in ionization energies, ΔIE, and the change in electron affinities, ΔEA, upon increasing the number of triply -F (panel (a)) and -CN (panel (b)) substituted benzenes in the periphery of the studied molecules (see Figure 1 of the main manuscript). While in Figure 3 of the main manuscript, ΔIE and ΔEA are calculated as energy differences between ions and neutral molecules, here they are approximated by orbital energies.

±0.5 eV; 8 × (3-F)''

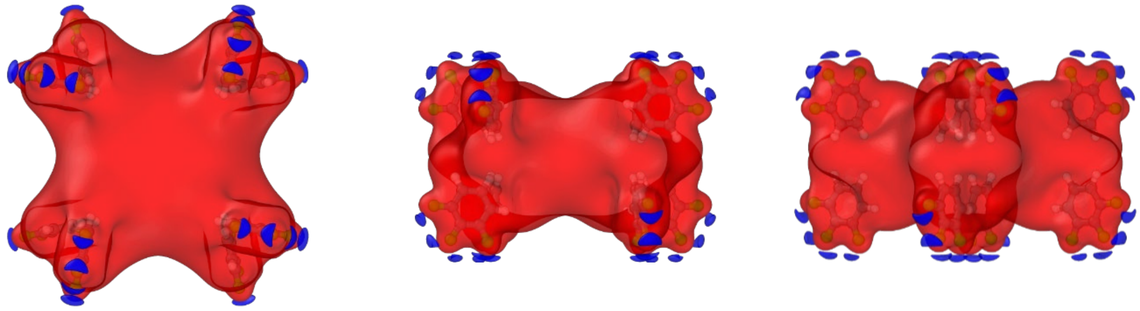

±0.3 eV; 8 × (3-F)''

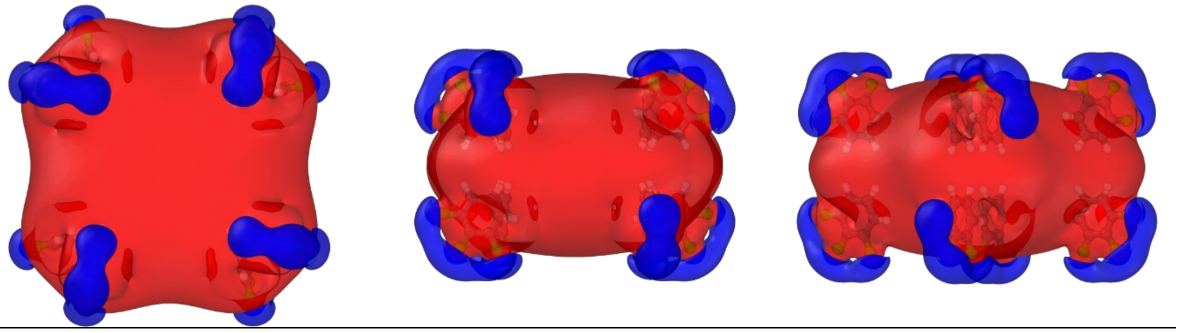

±0.5 eV; 6 × (3-F)''

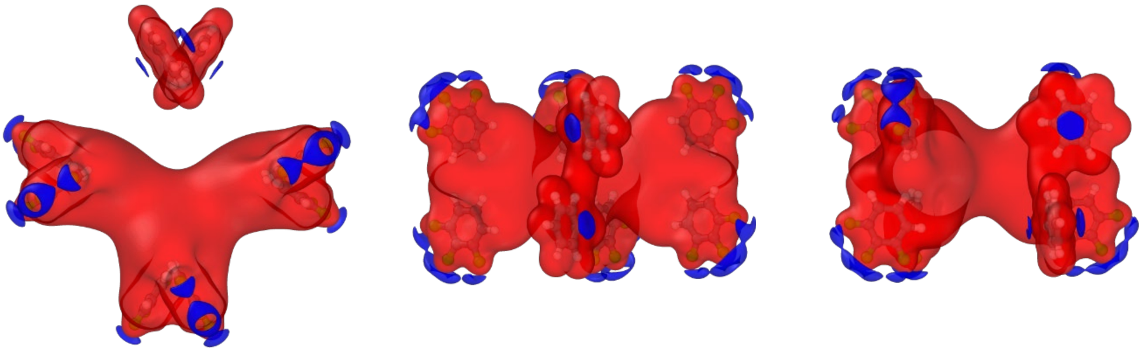

±0.3 eV; 6 × (3-F)''

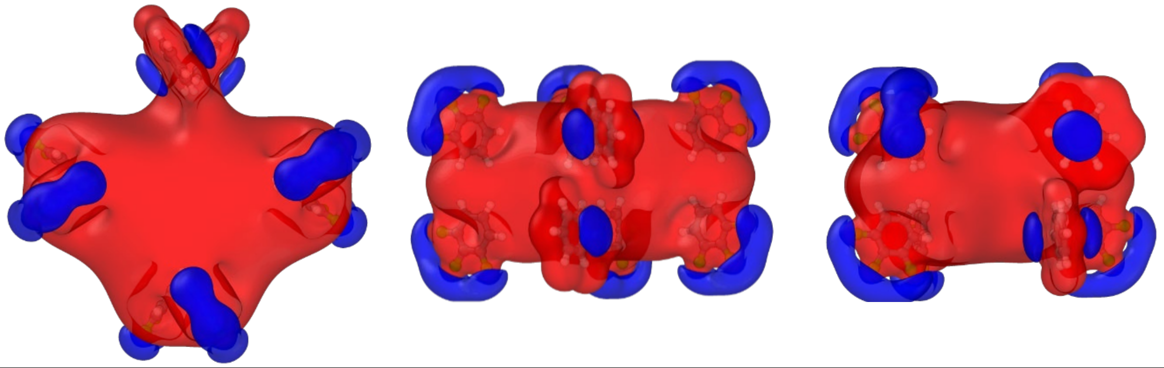

±0.5 eV; 4 × (3-F)''

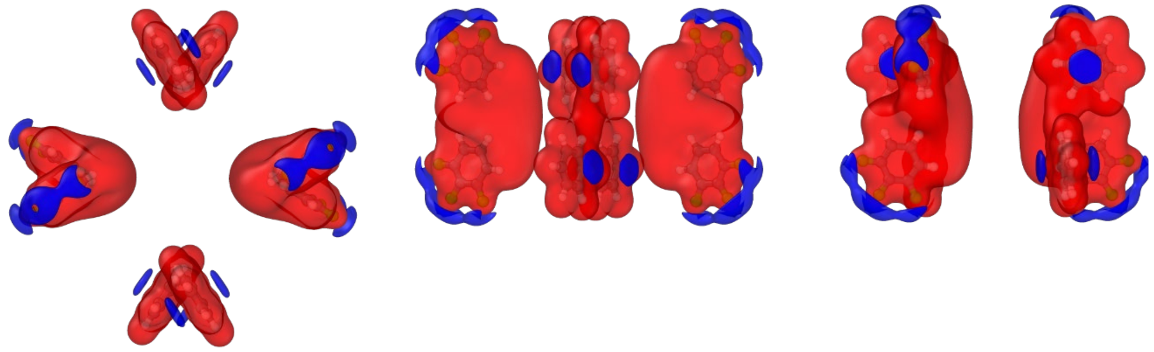

±0.3; eV 4 × (3-F)''

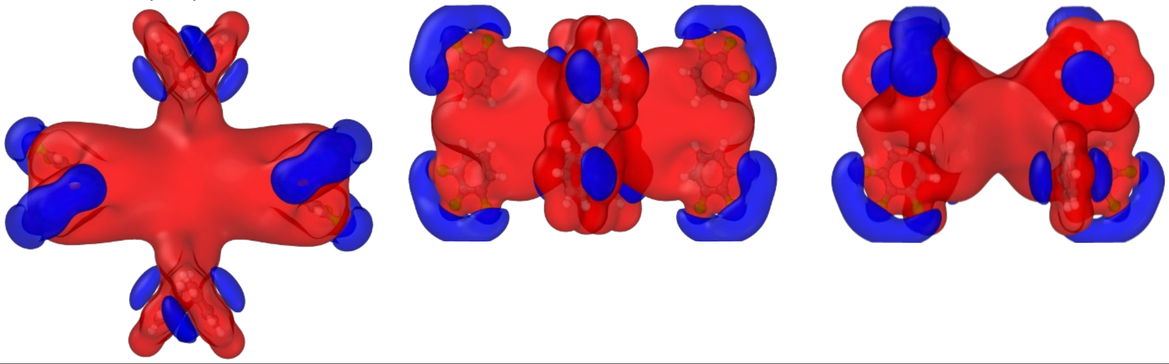

±0.5 eV; 2 × (3-F)''

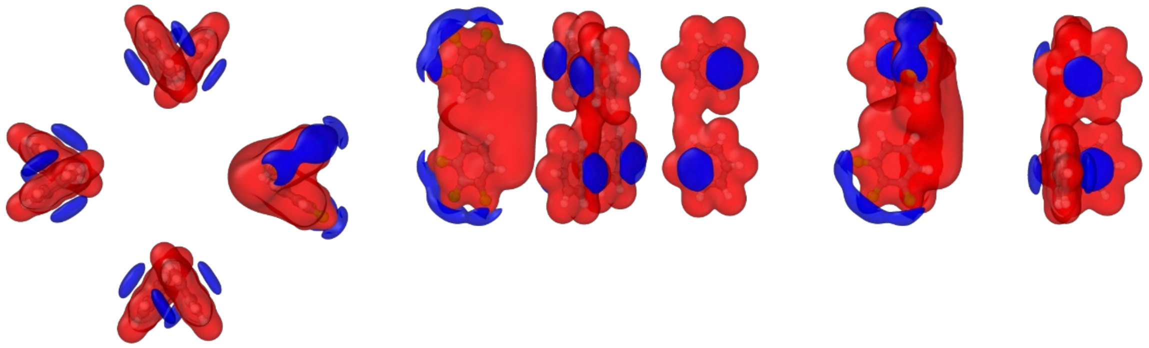

±0.3 eV; 2 × (3-F)''

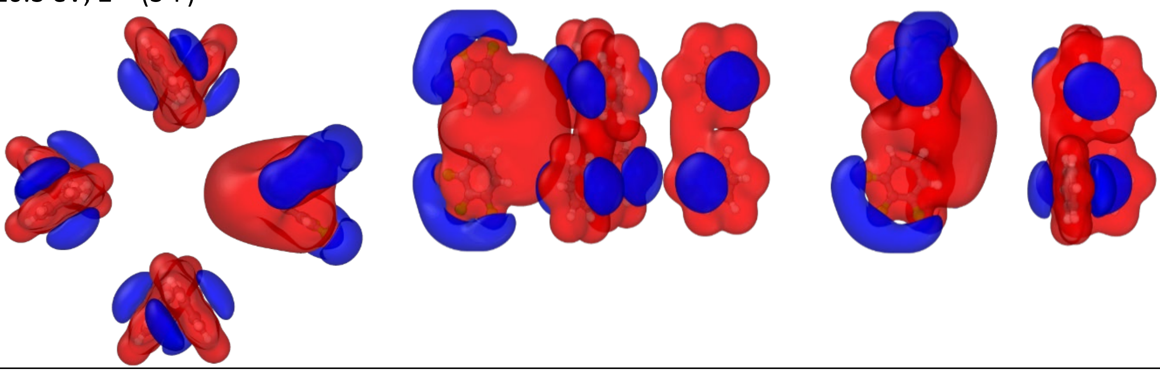

±0.5 eV; 1 × (3-F)“

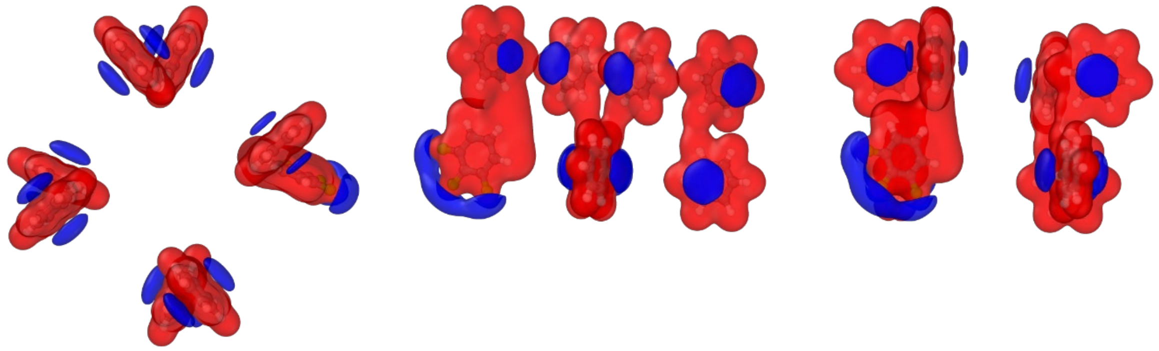

±0.3 eV; 1 × (3-F)“

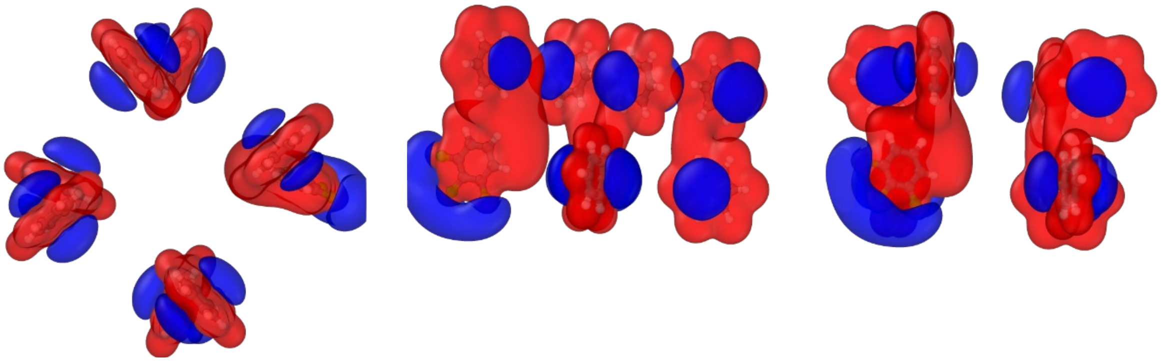

Figure S5: Isosurfaces (for two different pairs of isovalues) illustrating the electrostatic energy of an electron caused by the peripheral, substituted benzenes for the F-substituted systems for a decreasing degree of substitution (from top to bottom)

±1.0 eV; 8 × (3-CN)“

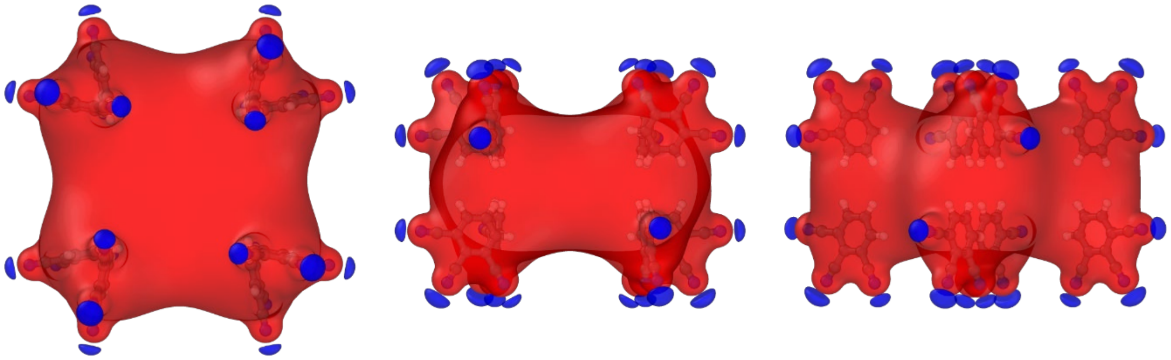

±0.8 eV; 8 × (3-CN)“

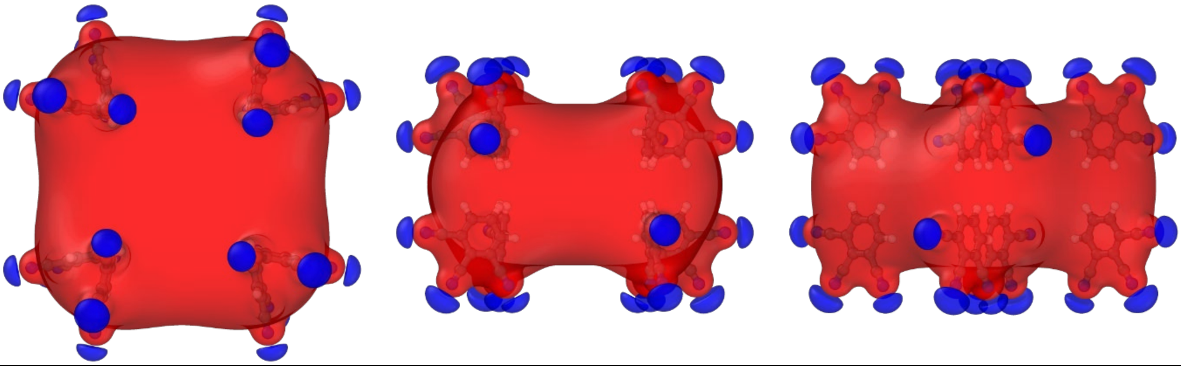

±1.0 eV; 6 × (3-CN)‘‘

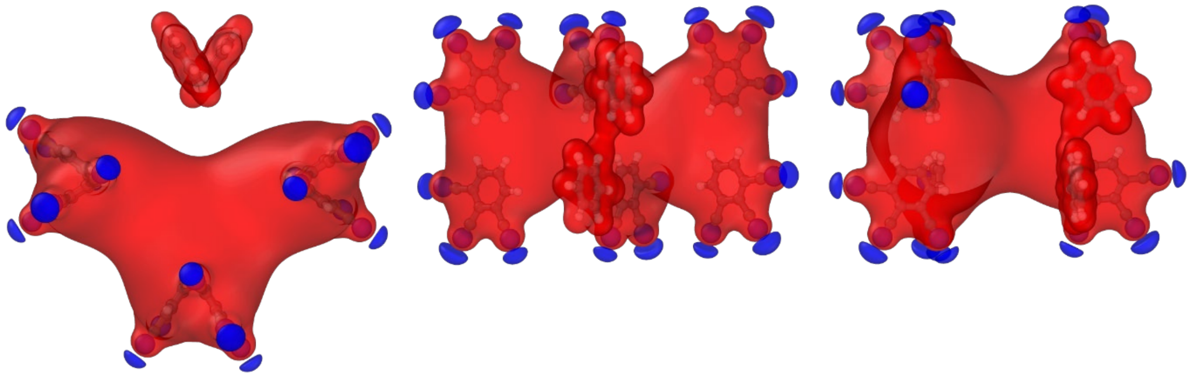

±0.8 eV; 6 × (3-CN)‘‘

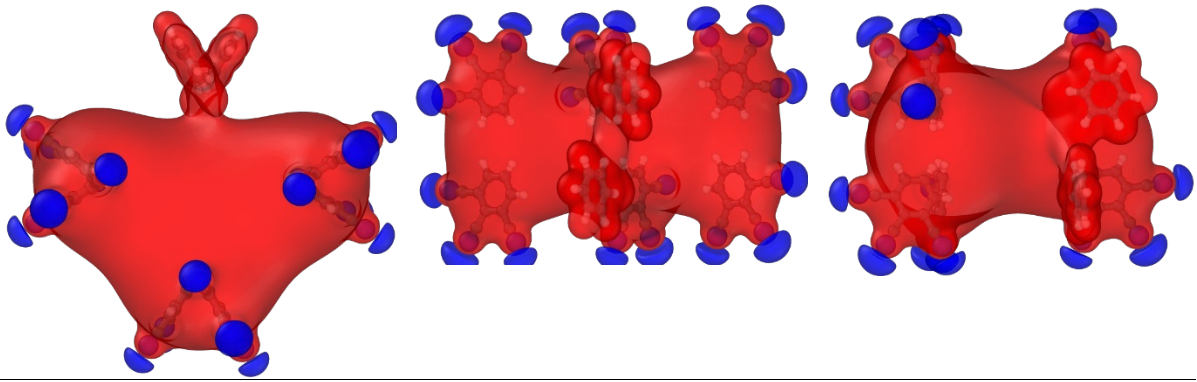

±1.0 eV; 4 × (3-CN)‘‘

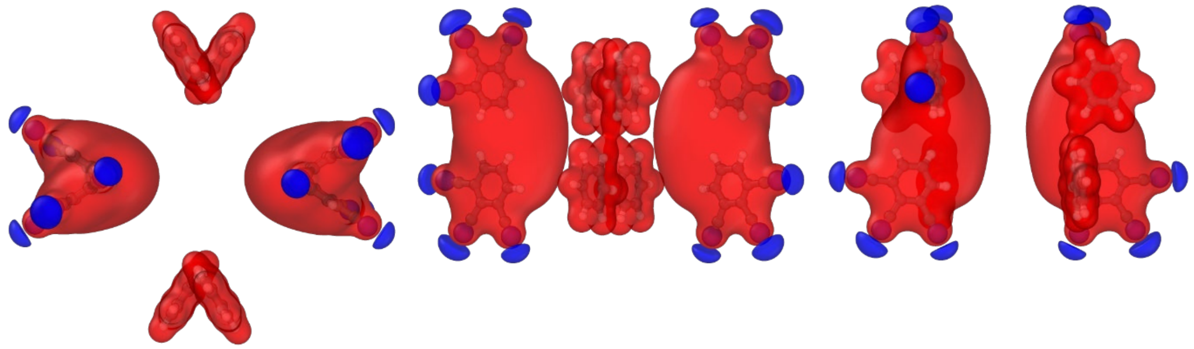

±0.8 eV; 4 × (3-CN)‘‘

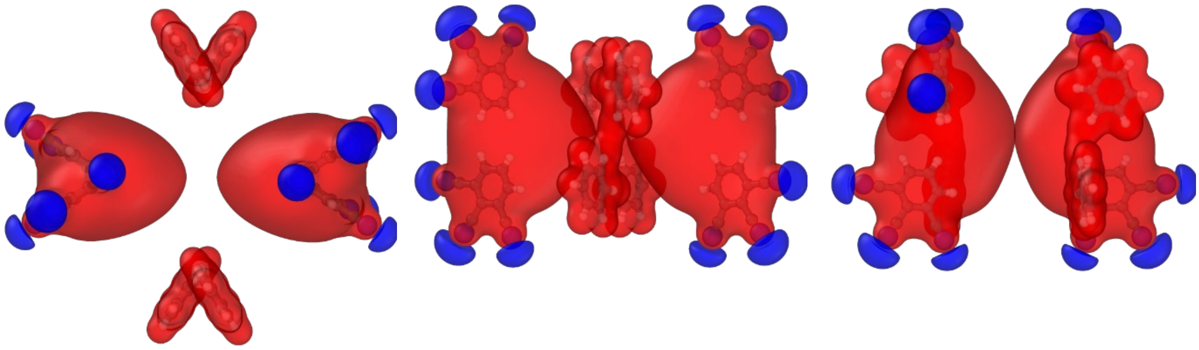

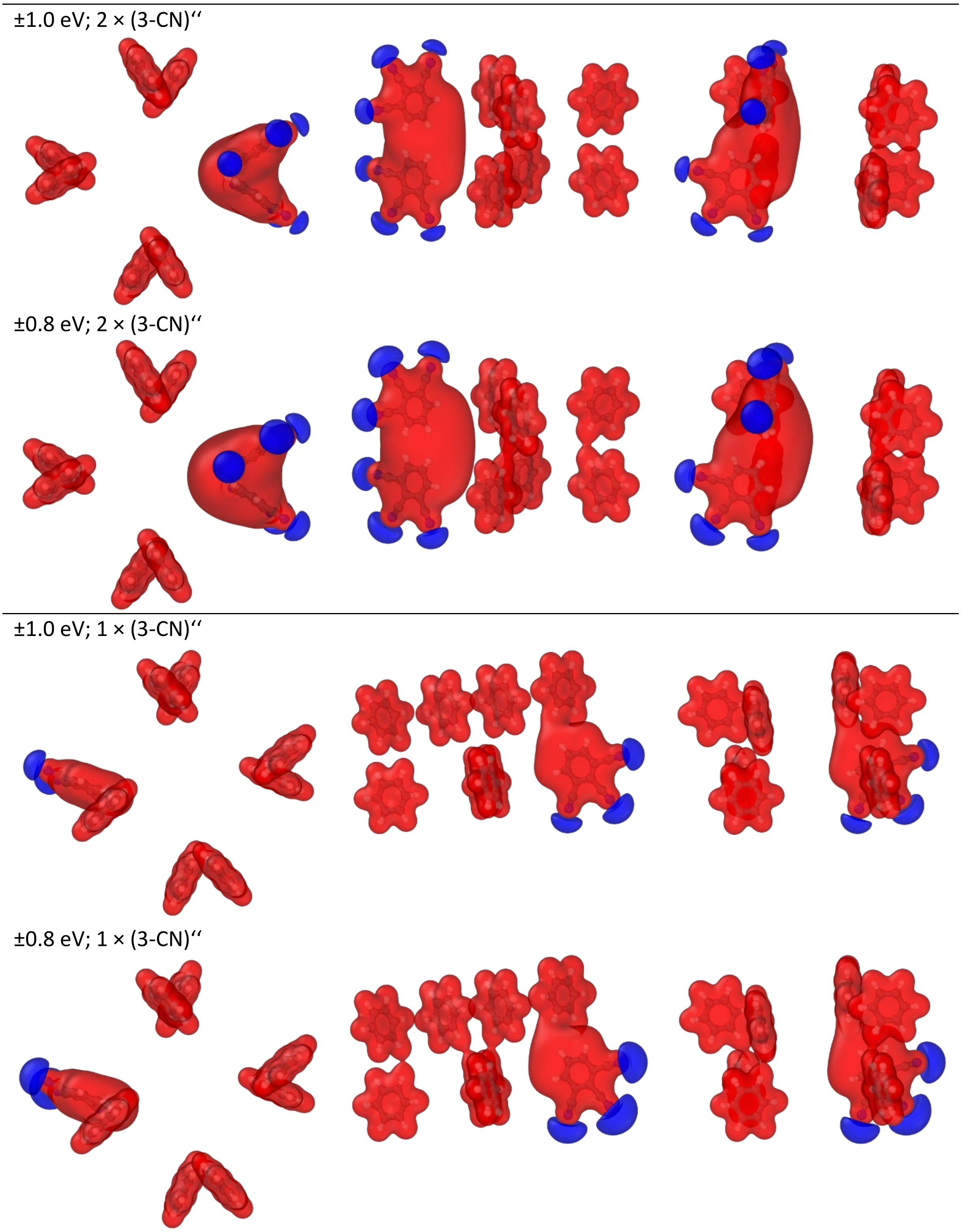

Figure S6: Isosurfaces (for two different pairs of isovalues) illustrating the electrostatic energy of an electron caused by the peripheral, substituted benzenes for the CN-substituted systems for a decreasing degree of substitution (from top to bottom)

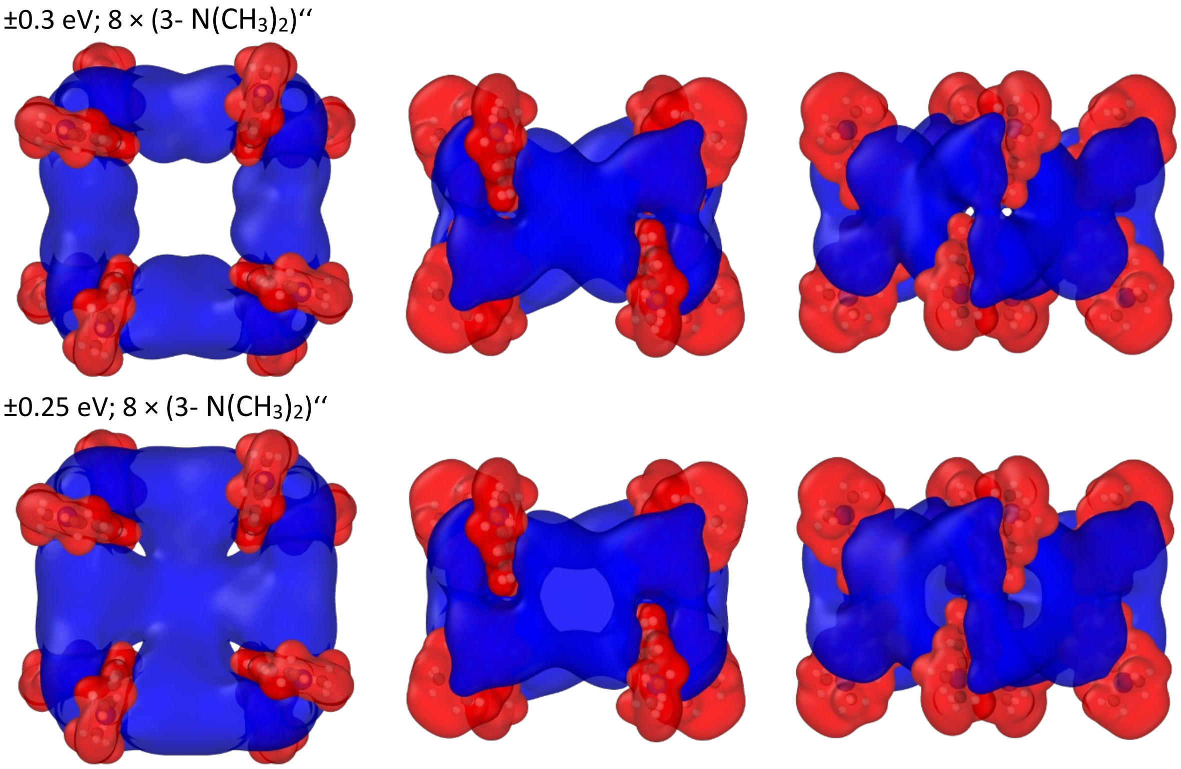

Figure S7: Isosurfaces (for two different pairs of isovalues) illustrating the electrostatic energy of an electron caused by the peripheral, fully $N(CH_3)_2$-substituted benzenes.
